# Spatial Memory and Gut Microbiota Alterations Are Already Present in Early Adulthood in a Pre-clinical Transgenic Model of Alzheimer’s Disease

**DOI:** 10.3389/fnins.2021.595583

**Published:** 2021-04-29

**Authors:** Paola C. Bello-Medina, Fernando Hernández-Quiroz, Marcel Pérez-Morales, Diego A. González-Franco, Guadalupe Cruz-Pauseno, Jaime García-Mena, Sofía Díaz-Cintra, Gustavo Pacheco-López

**Affiliations:** ^1^División de Ciencias Biológicas y de la Salud, Universidad Autónoma Metropolitana (UAM), Unidad Lerma, Lerma, Mexico; ^2^Departamento de Genética y Biología Molecular, Centro de Investigación y de Estudios Avanzados (CINVESTAV) del Instituto Politécnico Nacional (IPN), Unidad Zacatenco, Ciudad de México, Mexico; ^3^Doctorado en Ciencias Biológicas y de la Salud, Universidad Autónoma Metropolitana (UAM), Unidad Lerma, Lerma, Mexico; ^4^Departamento de Neurobiología del Desarrollo y Neurofisiología, Instituto de Neurobiología, Universidad Nacional Autónoma de México (UNAM), Querétaro, Mexico

**Keywords:** 3xTg-AD, dysbiosis, novel-object localization, Actinobacteria, TM7, alpha-diversity, beta-diversity, high-throughput DNA sequencing

## Abstract

The irreversible and progressive neurodegenerative Alzheimer’s disease (AD) is characterized by cognitive decline, extracellular β-amyloid peptide accumulation, and tau neurofibrillary tangles in the cortex and hippocampus. The triple-transgenic (3xTg) mouse model of AD presents memory impairment in several behavioral paradigms and histopathological alterations from 6 to 16 months old. Additionally, it seems that dysbiotic gut microbiota is present in both mouse models and patients of AD at the cognitive symptomatic stage. The present study aimed to assess spatial learning, memory retention, and gut microbiota alterations in an early adult stage of the 3xTg-AD mice as well as to explore its sexual dimorphism. We evaluated motor activity, novel-object localization training, and retention test as well as collected fecal samples to characterize relative abundance, alpha- and beta-diversity, and linear discriminant analysis (LDA) effect size (LEfSe) analysis in gut microbiota in both female and male 3xTg-AD mice, and controls [non-transgenic mice (NoTg)], at 3 and 5 months old. We found spatial memory deficits in female and male 3xTg-AD but no alteration neither during training nor in motor activity. Importantly, already at 3 months old, we observed decreased relative abundances of Actinobacteria and TM7 in 3xTg-AD compared to NoTg mice, while the beta diversity of gut microbiota was different in female and male 3xTg-AD mice in comparison to NoTg. Our results suggest that gut microbiota modifications in 3xTg-AD mice anticipate and thus could be causally related to cognitive decline already at the early adult age of AD. We propose that microbiota alterations may be used as an early and non-invasive diagnostic biomarker of AD.

## Introduction

Alzheimer’s disease (AD) is an age-related and neurodegenerative disorder characterized by β-amyloid plaques and tau neurofibrillary tangle formation (Serrano-Pozo et al., 2011; Martins et al., 2018) with a progressive decline in cognitive functions (Querfurth and Laferla, 2010; Serrano-Pozo et al., 2011). One model for the study of AD is the triple-transgenic mouse (3xTg-AD), which contains three mutations associated with familial AD (APP Swedish, MAPT P301L, and PSEN1 M146V mutations). In this pre-clinical model, extracellular Aβ-peptide accumulation within the hippocampus appears by 6 months old, and changes in tau occur at 12–15 months old; with hyperphosphorylated tau aggregates detected also in the hippocampus (Oddo et al., 2003). These changes are associated with cognitive impairment in several behavioral paradigms, such as elevated maze, object recognition memory, Morris water maze, and T-maze tasks (Oddo et al., 2003; Chen et al., 2013; Cantanelli et al., 2014; Morin et al., 2016; Davis et al., 2017; Wei et al., 2017; Bello-Medina et al., 2019a). Histopathological and cognitive alterations have been mainly reported in the symptomatic stage of AD, i.e., at 6–9 months old, while learning and memory deficit have not been observed in the pre-symptomatic stage (3–5 months old) (Guzmán-Ramos et al., 2012; Cantanelli et al., 2014). Furthermore, symptoms and pathological manifestations are stronger in female than in male mice in this AD pre-clinical 3xTg-AD model. For instance, in 3xTg-AD mice from 6 to 23 months old, Aβ peptide largely accumulates within the hippocampus and cortex of females compared to males (Hirata-Fukae et al., 2008; Carroll et al., 2010a; Creighton et al., 2019). Likewise, in comparison to males, 3xTg-AD female mice from 6 to 14 months old display worse performance in peak interval procedure, novel-object recognition (NOR) task, spontaneous alternation task, and Morris water maze (Clinton et al., 2007; Carroll et al., 2010a; Creighton et al., 2019; Gür et al., 2019).

Alzheimer’s disease etiology is still unknown, with multiple factors proposed as triggers of its development and contributors to its progression, such as oxidative stress (Sonnen et al., 2008; Chen and Zhong, 2014; Cheignon et al., 2018; Pohanka, 2018), lipid (Hamilton et al., 2015; Sah et al., 2017), and glucose metabolism alterations (Chen and Zhong, 2013), synaptic plasticity impairment (Min et al., 2013; Cantanelli et al., 2014; Guillot et al., 2016; Morin et al., 2016), and neuroinflammation (Cuello, 2017). Interestingly, environmental factors involved in the development of age-related disorders as is AD concur with increased intestinal permeability (Tran and Greenwood-Van Meerveld, 2013) and gut microbiota alterations (Syeda et al., 2018). Gut microbiota includes all microorganisms thriving within the intestine, which are functionally related to the host (Sampson and Mazmanian, 2015). To maintain host health, bacteria consortia should be in a dynamic balance between symbiotic, commensal, and pathogenic bacteria (Koboziev et al., 2014). In contrast, dysbiosis occurs when such homeostasis is lost (Degruttola et al., 2016). Gut microbiota contains Gram-positive and -negative bacteria, such as Firmicutes and Bacteroidetes, respectively. In this regard, a relevant component of the outer membrane of Gram-negative bacteria is lipopolysaccharide (Tlaskalová-Hogenová et al., 2004). This endotoxin travels from a leaky intestine, via the bloodstream, to cerebral regions, inducing inflammatory and microglia-mediated innate immune responses that are associated with Aβ oligomers, dimers, and monomers. In this context, microbiota metabolites and/or their components might be part of etiological factors of AD contributing to amyloid neurotoxicity (Colangelo et al., 2002; Dasari et al., 2011; Ferrera et al., 2014; Zhao and Lukiw, 2015) that results in the AD progression or its acceleration.

Currently, the gut microbiota is under characterization in several mouse pre-clinical models of AD to define pathological relationships and their potential causalities. In this context, most data focused on describing dysbiotic gut microbiota within the AD symptomatic stages. For instance, 5xFAD transgenic mice of 6 months old exhibit more abundance of the Proteobacteria and Firmicutes populations than control mice, while the Bacteroidetes population is lower (Lee et al., 2019). APPPS1 transgenic mice of 8 months old displayed significant proportion reductions in Firmicutes, Proteobacteria, and Actinobacteria, with increases in Bacteroidetes and Tenericutes phylum in comparison to wild-type (WT) mice. Regarding bacterial abundance, Rikenellaceae increases, while *Allobaculum* and *Akkermansia* genera decrease as compared to WT mice (Harach et al., 2017). Furthermore, in the 3xTg-AD mice at the symptomatic age of 9 months old, a significant increase in the abundance of Bacteroidetes and Firmicutes but a decrease of Cyanobacteria, Proteobacteria, Tenericutes, and Verrucomicrobia in comparison to WT mice were reported (Syeda et al., 2018).

However, so far, it is unknown if gut microbiota alterations precede cognitive deficits; therefore, we aimed to characterize gut microbiota at an early adulthood age in the 3xTg-AD mice. Furthermore, we analyzed whether sex might elicit dimorphism in the cognitive and gut microbiota parameters studied.

## Materials and Methods

The experiments reported in this study were carried out following the international (National Institutes of Health Guide for the Care and Use of Laboratory Animals, National Research Council, 2011) and the Mexican Official standard (NOM-036-SSA-2-2002) normative and were approved by the local ethics committee (Comité de Bioética del Instituto de Neurobiología, Universidad Nacional Autónoma de México), approval N° 117.A.

### Animals

The study subjects were female (*n* = 10) and male (*n* = 10) 3xTg-AD mice harboring APP_Swe_ and Tau_P__30__L_ transgenes on a mutant PS1_M__146__V_ knock-in background and female (*n* = 10) and male (*n* = 10) non-transgenic mice (NoTg) from the same genetic background B6129SF1/J (both Jackson Laboratory, Bar Harbor, ME, United States). B6129SF1/J hybrid mice are the offspring of the breeding between C57BL/6J females (B6) and 129S1/SvImJ males (129S). All mice were housed in groups of 3–5 per cage with water and food (LabDiet 5001) *ad libitum* and maintained in a room with 12-h dark/12-h artificial light cycles beginning at 19:00 h. We performed all behavioral procedures between 9:00 and 13:00 h.

### Genotyping

We performed genotyping as previously reported (Guzmán-Ramos et al., 2012). Briefly, for DNA extraction, a 5-mm-long caudal tail segment was sectioned, and lysis was made in an alkaline reagent (25 mM NaOH, 0.2 mM disodium EDTA) with heat (95°C, 1 h) and neutralization with a suitable buffer (1 M Tris–HCl at pH 7.5). This DNA sample was used immediately in a polymerase chain reaction (PCR) to test for the presence of the amyloid precursor protein (APP) and tau DNA and mutation in the presenilin 1 (PS1) gene.

### Collection of Fecal Samples for Microbiota Analysis

We collected fecal samples from NoTg and 3xTg-AD mice when they were 3 and 5 months old. Fresh fecal pellets were collected by holding the mouse in one hand while the mouse defecates directly in a 1.5 ml polypropylene tube held in the other hand; 7–8 fresh fecal pellets were gathered. Tubes were kept in dry ice until transfer to a −80°C freezer where the samples were maintained until their DNA microbiota profile analysis.

### High-Throughput V3-16S rDNA Libraries Sequencing

Fecal DNA was extracted from 100 mg of homogenized feces using Favor prep stool kit (Cat. #FASTI001-1; Favorgen Biotech Corp., Ping-Tung, Taiwan) according to the manufacturer’s instructions and stored at −70°C until sequencing (Hernández-Quiroz et al., 2020). We measured DNA concentration using a NanoDrop 2000 spectrophotometer (Thermo Fisher Scientific, Waltham, MA, United States), and DNA quality was evaluated by electrophoresis in 0.5% agarose gel. All PCR reactions were performed in a final volume in 50 μl final volume of 1× PCR buffer (50 mM KCl, 10 mM Tris–HCl, pH 8.8), 2 mM MgCl_2_, 0.2 μM of each barcoded primer, 0.2 mM each dNTP, 0.025 U/μl of recombinant Taq polymerase (Thermo Scientific EP0402), and 20–50 ng of total nucleic acids. The PCR program was 95°C, 5 min, followed by 30 × [94°C, 30 s; 62°C, 15 s; 72°C 15 s], 72°C, 10 min extension using a GeneAmp PCR System 2700 (Applied Biosystems). For library preparation, each of the 1–45 barcoded amplicons were quantified by gel densitometry and equal mass amounts pooled. The mixture was purified using E-Gel iBase Power System (Invitrogen). The library’s size and concentration were checked using the Agilent 2100 Bioanalyzer system and High Sensitivity DNA Kit (Agilent, United States). High-throughput sequencing of the ∼281-bp amplicons was performed using Ion OneTouch^TM^ 2, Ion PGM^TM^ Template OT2 200 Kit v2 DL (Life Technologies, Carlsbad, CA, United States), Ion 318 Chip Kit v2, and Ion Torrent PGM System (García-Mena et al., 2016). The amplicon was not observed for the negative controls and was not sequenced. After sequencing, reads were filtered by the PGM software to exclude low-quality and polyclonal sequences. All reads were analyzed using FastQC software v0.11.9 (Andrews, 2010) and trimmed to 200 nt using Trimmomatic v0.38. The sequencing summary is shown in Table 1. Demultiplexed FASTQ files were converted into FASTA files, concatenated into a single file, and then processed with multiple Quantitative Insights Into Microbial Ecology (QIIME) v1.9.0 scripts (Caporaso et al., 2010). DNA sequences were classified into operational taxonomic units (OTUs) using closed-based picking parameters with a 97% similarity level against the Greengenes database v13.8.

**TABLE 1:** Sequencing summary after trimming^#^ (*n* = 81).

Parameter	Fecal samples, *n* = 81
Number of reads	5,189,402
Length mean^a^	142 bp (28.01)^b^
Reads mean^a^	42,705.63
Reads min–max^a^	2,950–237,259
Identified OTUs	5,840
Total OTUs count	2,195,820
Samples with <10,000 reads	13

*^#^Reads were analyzed using FastQC software v0.11.9 and trimmed to 200 nt using Trimmomatic v0.38. ^a^Length expressed as bases. ^b^Standard deviation, for all samples. OTUs, operational taxonomic units.*

The corresponding FASTQ sequence files for all samples used in this study were deposited in the NCBI BioProject repository (Accession Number: PRJNA648144). In link: https://www.ncbi.nlm.nih.gov/bioproject/PRJNA648144.

### Gut Microbiota Relative Abundance and Diversity

We analyzed sequenced data using QIIME (v1.9.0) pipeline (Caporaso et al., 2010) to determine the relative abundance of gut bacterial taxa. Before calculation of alpha diversity, the OTU table was rarefied at 10,000 sequences per sample using a “single_rarefaction.py” QIIME script. Diversity was characterized by alpha diversity, including Shannon, Simpson, Chao1 indexes, and observed species using phyloseq (v1.22.3) and ggplot2 (v3.1.0) packages in R (v3.4.4) (McMurdie and Holmes, 2013). The beta diversity dissimilarity index was calculated by UniFrac distance metric as a percentage of the total variability in different axis of the plot and visualized by principal coordinate analysis as described in Chávez-Carbajal et al. (2019).

### Analysis of Significant Enrichment in Gut Microbiota Taxa

We used linear discriminant analysis (LDA) effect size (LEfSe) (v1.0) to disclose significant differences in the relative abundance of bacterial taxa among groups. These LEfSe analyses are represented in the form of bar plots and the parameters set with default *p* < 0.05, LDA scores ≥ 2.0 (Segata et al., 2011).

### Novel-Object Localization (NOL) Task Apparatus

The apparatus was an open acrylic box (33 cm × 33 cm × 33 cm) with black walls. The floor of the box was covered with a 1-cm layer of sawdust, the box was cleaned with 7% acetic acid (v/v), and sawdust was replaced between trials. There were four visual cues on the walls of the experimental room, within the visual field of mice. A video camera was positioned above the box, and each trial was video recorded for post-training analysis. The objects used were glass scintillation vials of 2.8 cm × 6.1 cm; these stimuli were denominated familiar localization 1 (Fam 1) and familiar localization 2 (Fam 2), respectively. These objects were attached with Velcro^®^ to the box floor and were cleaned after each trial with 70% alcohol solution (v/v).

### Training and Retention Test

**FIGURE 1:**
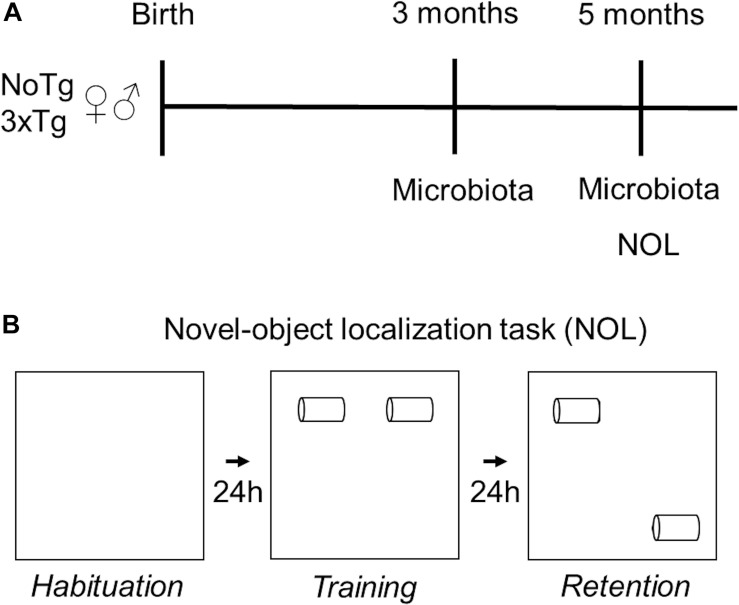
Experimental design. **(A)** Fecal samples of female and male non-transgenic (NoTg) or Alzheimer’s disease triple-transgenic (3xTg-AD) mice were collected when they were 3 and 5 months old. **(B)** NOL task was performed in 5-month-old mice. NOL consists of three sessions: habituation, training, and retention test.

Handling of 5-month-old mice took place for 5 min daily on three consecutive days. The NOL task consisted of three sessions: habituation, training, and retention. During the habituation session, the mice could freely explore the open box, without objects, for 5 min. In this session, motor activity parameters such as speed, distance traveled, and resting time were evaluated. Twenty-four hours after habituation, during the training session, the mice were placed in the open box, which contained the two-sample objects (Fam 1 and Fam 2), for 10 min. Retention took place 24 h after the training session; mice were placed in the open box where one of the familiar objects remained in the same localization it was located in training (Fam), and the other familiar object was moved to a novel localization (Nov) (Figure 1). The results were expressed both as total exploration time per object.

### Immunohistochemistry for β-Amyloid Peptide Detection

After NOL retention, and under anesthesia, we euthanized the mice (*n* = 4 per group) and transcardially perfused 4% paraformaldehyde in 0.1 M phosphate buffer via the ascending aorta. Brains were removed, postfixed in the same solution overnight. They were cryoprotected with 30% sucrose in phosphate buffer for 6 days. Four frozen sagittal sections of 30 μm from the left hemisphere that contained the *subiculum* and CA1 of the dorsal hippocampus (lateral from 0.72 to 1.80 mm to interhemispheric line; Paxinos and Franklin, 2001) were cut with the aid of a Leica cryostat and were placed on slides. Sections on slides were rinsed in phosphate-buffered saline (PBS) and incubated in 90% formic acid for 7 min and rinsed with water after were incubated in 1% H_2_O_2_, and then washed with PBS. After blocking with a tyramide signal amplification kit (TSA) blocking buffer (PerkinElmer Life Sciences), the sections were incubated in a monoclonal mouse anti-BAM10 antibody (1:500; Sigma-Aldrich) overnight at 4°C. Subsequently, sections were washed with PBS and incubated for 2 h at room temperature with Alexa Fluor 488-coupled goat anti-mouse antibody (1:500; Life Technologies), then washed with PBS, and the nuclei were counterstained with 4′,6-diamidino-2-phenylindole (DAPI) (1:5,000; Sigma-Aldrich). The stained sections were covered with fluorescence mounting medium (Fluoromount-G, Electron Microscopy Sciences).

The subiculum and CA1 mosaic images used in the analyses were obtained with a 40×/1.25 apochromatic objective lens and the MosaiX module for the Apotome system (Zeiss). Analysis of the β-amyloid accumulation was performed on the images using the ImageJ software, the proceeding was carried out as described previously (González-Franco et al., 2017; Prado-Alcalá et al., 2017). The results were expressed as the area that is occupied for β-amyloid in the subiculum or CA1 of the dorsal hippocampus.

### Statistical Analysis

We performed the Kolmogorov–Smirnov test to prove the normality parametric assumption. The data analysis of motor activity was made with a two-way ANOVA, where factor 1 was genotype (NoTg or 3xTg-AD) and factor 2 was sex (female or male). The data analysis of NOL performance was made with a three-way ANOVA, where factor 1 was genotype (NoTg or 3xTg-AD), factor 2 was sex (female or male), and within-subject factor 3 was object localization (Fam 1 vs. Fam 2 in training or Fam vs. Nov in the retention test). For the β-amyloid histological analysis, we applied a two-way ANOVA, where factor 1 was genotype (NoTg or 3xTg-AD) and factor 2 was sex (female or male) for each dorsal hippocampus area (*subiculum* or CA1). We used the *post hoc* Bonferroni test when appropriate. The *p* < 0.05 was considered statistically significant.

For gut microbiota analyses, all data were statistically analyzed using *t*-test or Mann–Whitney *U* test. Data were expressed in means ± standard deviation, the *p* < 0.05 was considered statistically significant. Sequenced data were analyzed using QIIME pipeline (v1.9.0). OTU picking was made against the Greengenes (v13.8) database. Bioinformatic analyses were made in R environment (v3.4.4); gut bacterial diversity (alpha diversity) was assessed with phyloseq (v1.22.3). Images were plotted using ggplot2 (v3.1.0) and RColorBrewer (v1.1-2) packages.

## Results

### Motor Activity

**FIGURE 2:**
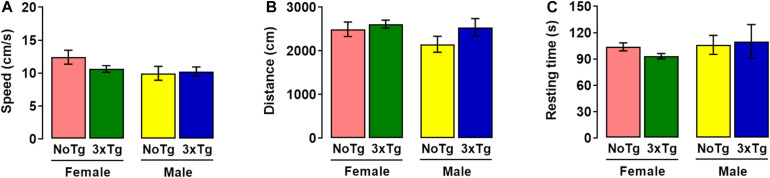
Motor activity during habituation session of novel-object localization (NOL) task. Mean values (with standard error) of **(A)** speed, **(B)** distance traveled, and **(C)** resting time of female or male non-transgenic (NoTg) or Alzheimer’s disease triple-transgenic (3xTg-AD) mice of 5 months old; *n* = 10 mice per group.

In habituation session of NOL, two-way ANOVA showed no differences in speed for genotype [*F*_(__1_,_36__)_ = 0.77; *p* = 0.38], sex [*F*_(__1_,_36__)_ = 2.71; *p* = 0.11], nor interaction [*F*_(__1_,_36__)_ = 1.42; *p* = 0.24] factors (Figure 2A). Two-way ANOVA showed no alterations in traveled distance for genotype [*F*_(__1_,_36__)_ = 2.35; *p* = 0.13], sex [*F*_(__1_,_36__)_ = 1.60; *p* = 0.21], nor interaction [*F*_(__1_,_36__)_ = 0.66; *p* = 0.42] factors (Figure 2B). The two-way ANOVA showed no significant differences in resting time and showed no statistical differences in genotype [*F*_(__1_,_36__)_ = 0.08; *p* = 0.77], sex [*F*_(__1_,_36__)_ = 0.72; *p* = 0.40], nor with interaction [*F*_(__1_,_36__)_ = 0.42; *p* = 0.52] factors (Figure 2C).

### Training of NOL

**FIGURE 3:**
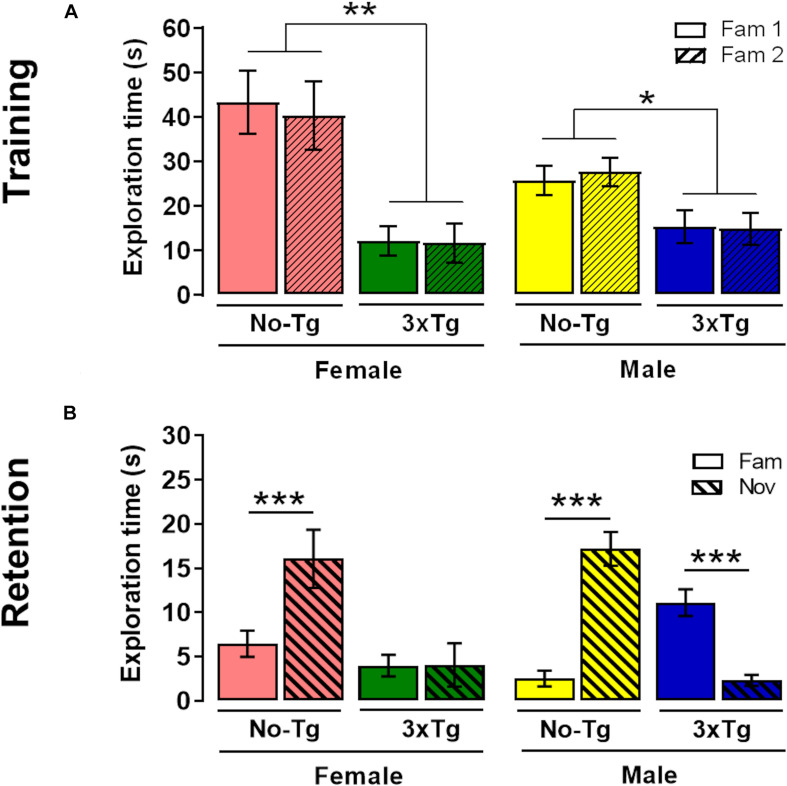
Novel-object localization (NOL) task. Mean exploration time (with standard error) of familiar localization 1 (Fam 1) and familiar localization 2 (Fam 2) in **(A)** training and exploration time of familiar localization (Fam) and novel localization (Nov) in **(B)** retention test of female or male non-transgenic (NoTg) or Alzheimer’s disease triple-transgenic (3xTg-AD) mice of 5 months old. *p < 0.05, **p < 0.001, ***p < 0.0001; *n* = 10 mice per group.

A three-way ANOVA showed a significant effect in total time object exploration during training for genotype [*F*_(__1_,_72__)_ = 36.63; *p* < 0.0001], but not for object [*F*_(__1_,_72__)_ = 0.03; *p* = 0.87] nor sex [*F*_(__1_,_72__)_ = 3.00; *p* = 0.08] factors. The genotype × sex [*F*_(__1_,_72__)_ = 0.02; *p* = 0.87], genotype × object [*F*_(__1_,_72__)_ = 2.18 × 10^–4^; *p* = 0.99], sex × object [*F*_(__1_,_72__)_ = 0.12; *p* = 0.73], and genotype × sex × object [*F*_(__1_,_72__)_ = 0.13; *p* = 0.72] interactions were not statistically significant (Figure 3A). The *post hoc* Bonferroni test showed that exploration time was higher in Fam 1 object in female NoTg than female 3xTg-AD (*p* = 0.001) and Fam 2 object in female NoTg in comparison with female 3xTg-AD (*p* = 0.005) (Figure 3A). The same effect was observed in male NoTg vs. 3xTg-AD in Fam 1 (*p* = 0.04) and Fam 2 (*p* = 0.01) objects (Figure 3A). The *post hoc* Bonferroni test showed that the exploration times for Fam 1 and Fam 2 were not significantly different in each genotype group; this result reflects a good familiarization process, which is very relevant for the acquisition of the recognition memory.

### Retention Test of NOL

In the retention test, a three-way ANOVA showed significant differences in the genotype [*F*_(__1_,_72__)_ = 15.56; *p* = 0.0002], object [*F*_(__1_,_72__)_ = 8.69; *p* = 0.004], but not in sex [*F*_(__1_,_72__)_ = 0.24; *p* = 0.63] factors. The genotype × object [*F*_(__1_,_72__)_ = 39.26; *p* < 0.0001] and genotype × sex × object [*F*_(__1_,_72__)_ = 6.98; *p* = 0.010] interactions were statistically significant; however, the genotype × sex [*F*_(__1_,_72__)_ = 2.41; *p* = 0.13] and sex × object [*F*_(__1_,_72__)_ = 0.52; *p* = 0.47] interactions were not significant. The *post hoc* Bonferroni test showed that exploration time NOL was higher than familiar-object place in female (*p* < 0.0001) and male (*p* < 0.0001) NoTg mice (Figure 3B). These results indicate a preference for the displaced object, which is a normal behavior for memory recognition (Bello-Medina et al., 2019b). The effect observed in female NoTg mice was opposite in male 3xTg-AD mice (*p* < 0.0001). No differences were found in exploration time between Nov and Fam objects in female 3xTg-AD mice. This demonstrated that there is a deficit in NOL memory in female and male 3xTg-AD mice. On the other hand, the Bonferroni test showed statistical differences in exploration time of object displaced in female NoTg and female 3xTg-AD. This same effect was also observed in male NoTg in comparison with male 3xTg-AD (Figure 3B).

### β-Amyloid Peptide Increases in the *Subiculum* and CA1 of the Dorsal Hippocampus in 3xTg-AD Mice

**FIGURE 4:**
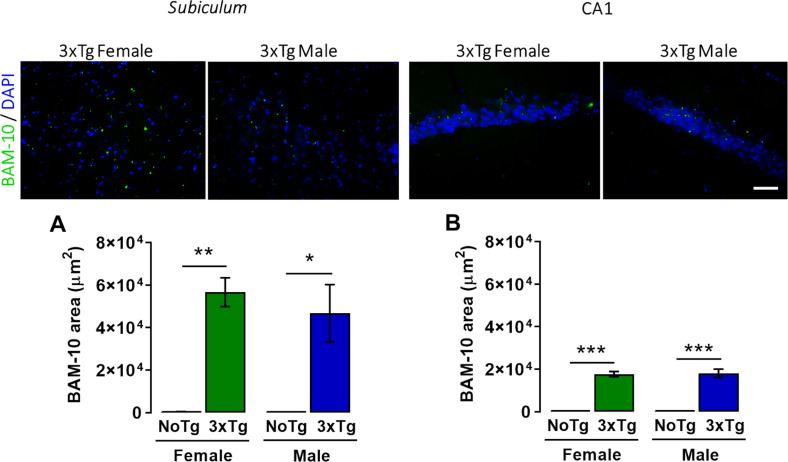
Representative images of β-amyloid (green) immunohistochemistry and nuclei detection (blue) obtained with 40× objective in the *subiculum* and CA1 of the dorsal hippocampus of female and male 3xTg-AD at 5 months old. Mean BAM-10 area (μm^2^) (with standard error) of female and male non-transgenic (NoTg) or Alzheimer’s disease triple-transgenic (3xTg-AD) mice in the **(A)** *subiculum* and **(B)** CA1. *p < 0.05, **p < 0.001, ***p < 0.0001; *n* = 4 mice per group. Scale bar = 20 μm.

A two-way ANOVA showed significant differences in area occupied by β-amyloid peptide in the *subiculum* for genotype [*F*_(__1_,_12__)_ = 45.94; *p* < 0.0001], but neither for sex [*F*_(__1_,_12__)_ = 0.44; *p* = 0.52] nor interaction [*F*_(__1_,_12__)_ = 0.42; *p* = 0.53] (Figure 4A). The *post hoc* Bonferroni test showed that the β-amyloid area was larger in female 3xTg-AD (14.7%) than female NoTg mice (0.28%) (*p* = 0.0002); this same effect was observed in male 3xTg-AD (13.4%) with respect to male NoTg (0.27%) (*p* = 0.014) (Figure 4A).

A two-way ANOVA showed statistical differences in the area occupied for β-amyloid peptide in the CA1 for genotype [*F*_(__1_,_12__)_ = 234.48; *p* < 0.0001] but not for sex [*F*_(__1_,_12__)_ = 0.29; *p* = 0.87] nor interaction [*F*_(__1_,_12__)_ = 0.29; *p* = 0.86] factors (Figure 4B). The *post hoc* Bonferroni test showed that the β-amyloid area was larger in female 3xTg-AD (56,705.1 μm^2^, which represent 10.9% of the total analyzed area of the *subiculum*) than female NoTg mice (344.9 μm^2^, which represent 10.9% of the total analyzed area of the *subiculum* 0.24%) (*p* = 0.0002); this same effect was observed in male 3xTg-AD (46,748.1 μm^2^, which represent 9.6% of the total analyzed area of the CA1) vs. male NoTg (257.77 μm^2^, which represent 0.23% of the total analyzed area of the CA1) (*p* = 0.014) (Figure 4B). These results mean that female and 3xTg-AD mice at 5 months old present β-amyloid accumulation in the *subiculum* and CA1 of the dorsal hippocampus at the early pre-symptomatic stage of AD.

### Changes in the Fecal Bacterial Diversity Relative Abundance of Bacterial Phyla Are Associated With the Triple Transgenic Condition in the Mouse Model

**FIGURE 5:**
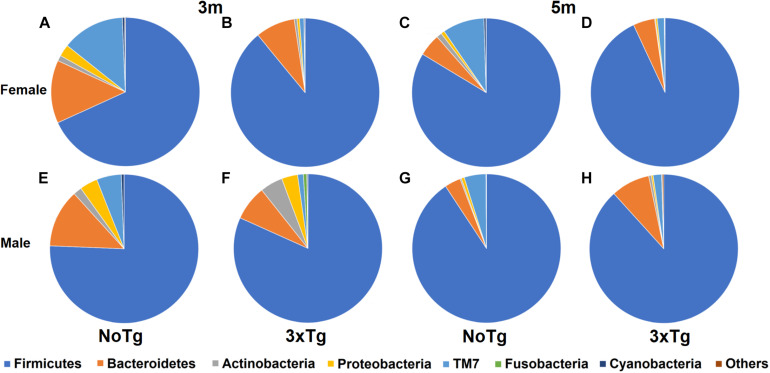
Relative abundance of bacterial phyla in fecal samples of non-transgenic (NoTg) and Alzheimer’s disease triple-transgenic (3xTg-AD) mice. Sectors in pie charts indicate Firmicutes, Bacteroidetes, Actinobacteria, Proteobacteria, TM7, Fusobacteria, and Cyanobacteria phyla as shown by tag colors at the right side of the figure. Abundances of each phylum are shown as percentages beside each sector in the pie charts. “Others” groups phyla with <0.5% relative abundance (Verrucomicrobia, Acidobacteria, Thermotogae, Chloroflexi, Thermi, Gemmatimonadetes, Tenericutes, Euryarchaeota, FBP, Spirochaetes, and Synergistetes). The figure shows data for 3 and 5 months (m) old for NoTg female **(A,C)** and male mice **(E,G)** and for 3xTg-AD female **(B,D)** and male mice **(F,H)** (Supplementary Tables 1, 2).

The characterization of the relative abundance of phyla in the fecal samples showed significant changes when comparing by age (3 vs. 5 months old); in the male NoTg mice, there was an increase in the abundance of Firmicutes (*p* = 0.017) and Actinobacteria (*p* = 0.005), while a decrease was detected for Proteobacteria (*p* = 0.004), Fusobacteria (*p* = 0.011), Cyanobacteria (*p* = 0.001), and Bacteroidetes (*p* = 0.008). A decrease in Actinobacteria (*p* = 0.049) in female 3xTg-AD mice was observed (Figure 5 and Supplementary Table 1). Concerning the genotype, the change in the abundance of TM7 was statistically significant. For instance, at 3 months, TM7 was more abundant in NoTg male mice vs. 3xTg-AD male (*p* = 0.003) and in NoTg female mice vs. 3xTg-AD female (*p* = 0.002). At 5 months, TM7 was more abundant in NoTg female mice vs. 3xTg-AD female (*p* = 0.007), and in males, this phylum was more abundant in NoTg male mice vs. 3xTg-AD male (*p* = 0.007). Fusobacteria were more abundant in 3xTg-AD male vs. NoTg male (*p* = 0.003), and Cyanobacteria were more abundant in NoTg male vs. 3xTg-AD male (*p* = 0.049) (Figure 5 and Supplementary Tables 1, 2). Finally, when comparing by sex, Cyanobacteria were more abundant at 3 months in NoTg male vs. NoTg female (*p* = 0.007), and Fusobacteria were more abundant at 3 months in 3xTg-AD male vs. 3xTg-AD female at 3 months (*p* = 0.031) (Figure 5 and Supplementary Tables 1, 2).

### Alpha and Beta Diversity of Gut Microbiota

**FIGURE 6:**
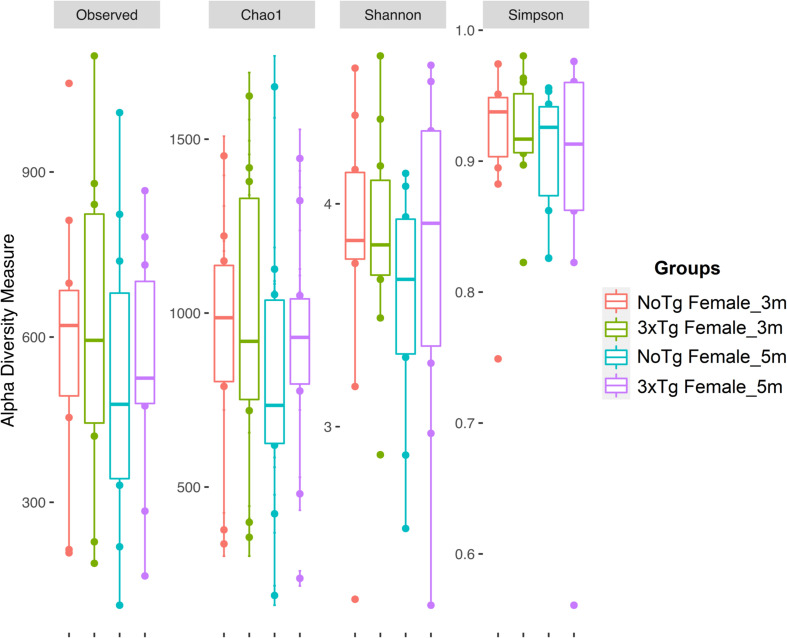
Alpha diversity of bacteria in fecal samples collected from female mice. The figure shows data for non-transgenic (NoTg) (coral pink) and Alzheimer’s disease triple-transgenic (3xTg-AD) mice at 3 months (m) old (light olive) and NoTg (light blue) and 3xTg-AD mice at 5 months old (light purple). The *Y*-axis indicates the values for the corresponding indexes: observed number of species (Observed), expected bacterial richness (Chao1), and Shannon and Simpson diversity (Supplementary Tables 3, 4).

**FIGURE 7:**
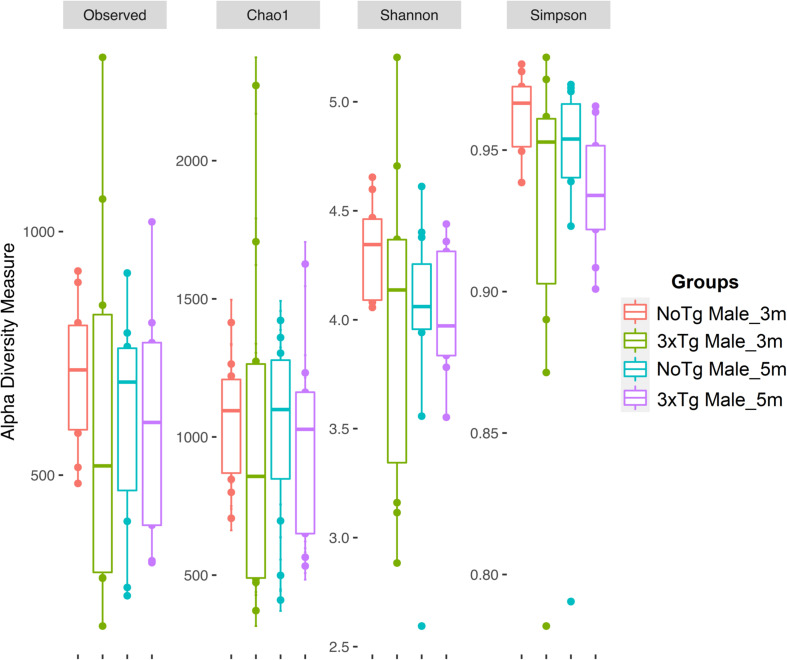
Alpha diversity of bacteria in fecal samples collected from male mice. The figure shows data for non-transgenic (NoTg) (coral pink) and Alzheimer’s disease triple-transgenic (3xTg-AD) mice at 3 months (m) old (light olive) and NoTg (light blue) and 3xTg-AD mice at 5 months old (light purple). The *Y*-axis indicates the values for the corresponding indexes: observed number of species (Observed), expected bacterial richness (Chao1), and Shannon and Simpson diversity (Supplementary Tables 3, 4).

The estimation of alpha diversities for the same data showed only statistically significant changes for Shannon (*p* = 0.041) and Simpson (*p* = 0.035) indexes when comparing NoTg female vs. NoTg male mice at 3 months and NoTg female vs. NoTg male mice at 5 months (Figures 6, 7 and Supplementary Table 4).

**FIGURE 8:**
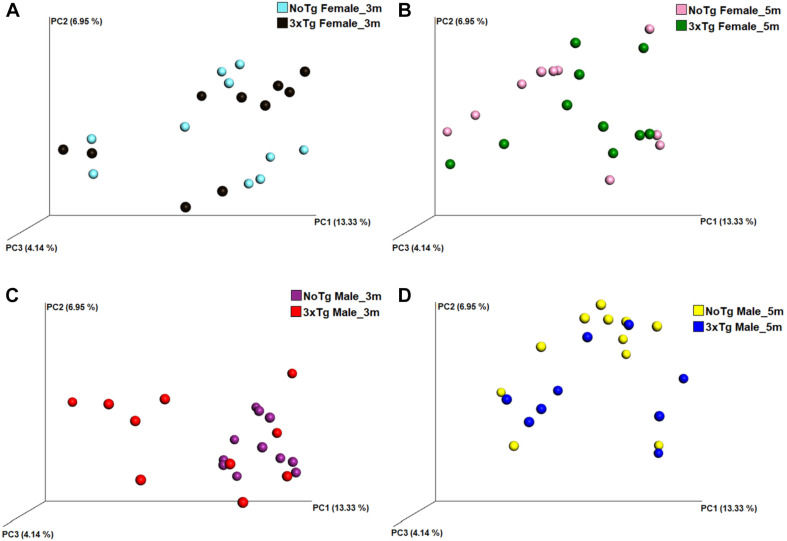
Beta diversity of bacteria in fecal samples collected from non-transgenic (NoTg) and Alzheimer’s disease triple-transgenic (3xTg-AD) mice. The graphics show beta diversity analyses calculated by dissimilarity metrics using operational taxonomic unit (OTU) tables and Unweighted UniFrac analyses. The analyses show the dissimilarity among mice by colors: NoTg female at 3 months (m) old (cyan), 3xTg-AD female at 3 months old (black), NoTg female at 5 months old (pink), 3xTg-AD female at 5 months old (green), NoTg male at 3 months old (purple), 3xTg-AD male at 3 months old (red), NoTg male at 5 months old (yellow), 3xTg-AD male at 3 months old (blue). Data comparisons by genotype and sex: NoTg vs. 3xTg-AD female mice at 3 months old **(A)**, NoTg vs. 3xTg-AD female mice at 5 months old **(B)**, NoTg vs. 3xTg-AD male mice at 3 months old **(C)**, and NoTg vs. 3xTg-AD male mice at 5 months old **(D)**. The three-dimensional scatter plots were generated using principal coordinates analyses (PCoA) in three different axis that show the percentage of total differences. There were significant differences on all comparisons according to ANOSIM similarity test (**(A)**, *R* = 0.184, *p* = 0.018; **(B)**, *R* = 0.124, *p* = 0.028; **(C)**, *R* = 0.422, *p* = 0.001; **(D)**, *R* = 0.174, *p* = 0.022).

**FIGURE 9:**
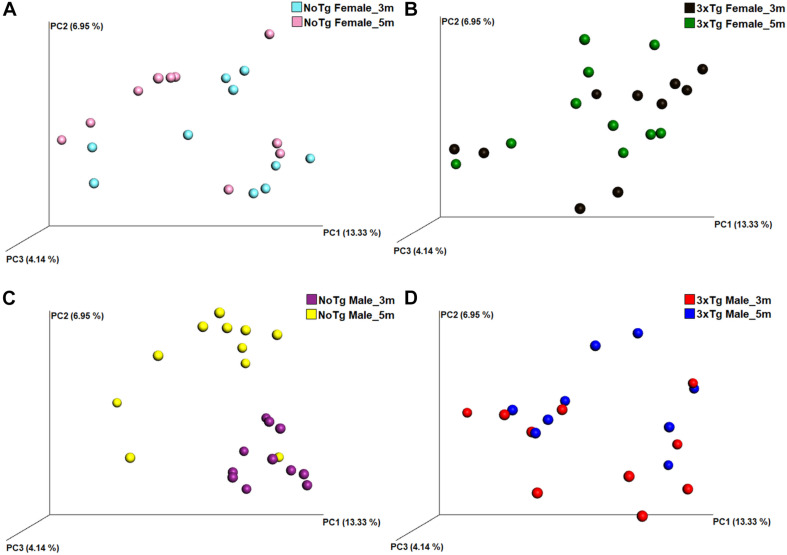
Beta diversity of bacteria in fecal samples collected from non-transgenic (NoTg) and Alzheimer’s disease triple-transgenic (3xTg-AD) mice. The graphics show beta diversity analyses calculated by dissimilarity metrics using operational taxonomic unit (OTU) tables and Unweighted UniFrac analyses. The analyses show the dissimilarity among mice by colors: NoTg female at 3 months (m) old (cyan), 3xTg-AD female at 3 months old (black), NoTg female at 5 months old (pink), 3xTg-AD female at 5 months old (green), NoTg male at 3 months old (purple), 3xTg-AD male at 3 months old (red), NoTg male at 5 months old (yellow), 3xTg-AD male at 3 months old (blue). Data comparisons by time in the same genotype: NoTg female mice 3 and 5 months old **(A)**, 3xTg-AD female mice 3 and 5 months old **(B)**, NoTg male mice 3 and 5 months old **(C)**, and 3xTg-AD male mice 3 and 5 months old **(D)**. The three-dimensional scatter plots were generated using principal coordinates analyses (PCoA) in three different axis that show the percentage of total differences. There is a significant difference in panel **(C)** according to ANOSIM similarity test (*R* = 0.425, *p* = 0.002) but not in panels **(A)** (*R* = 0.032, *p* = 0.223), **(B)** (*R* = 0.020, *p* = 0.297), and **(D)** (*R* = 0.093, *p* = 0.076).

**FIGURE 10:**
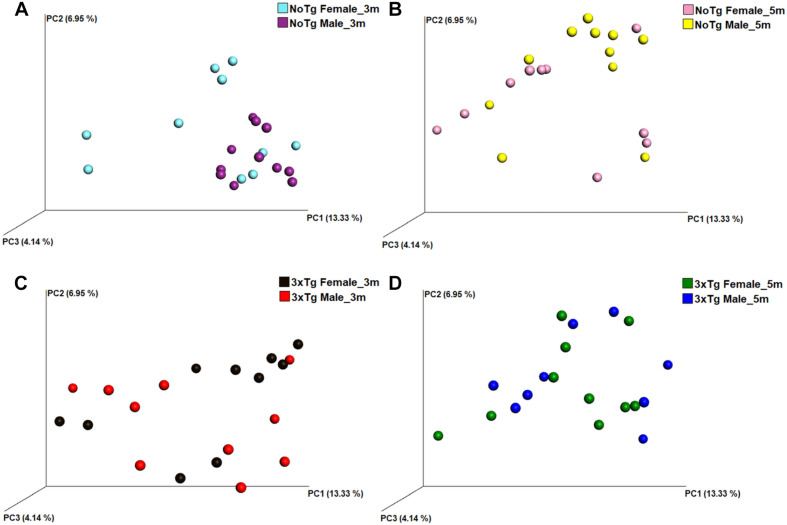
Beta diversity of bacteria in fecal samples collected from non-transgenic (NoTg) and Alzheimer’s disease triple-transgenic (3xTg-AD) mice. The graphics show beta diversity analyses calculated by dissimilarity metrics using operational taxonomic unit (OTU) tables and Unweighted UniFrac analyses. The analyses show the dissimilarity among mice by colors: NoTg female at 3 months (m) old (cyan), 3xTg-AD female at 3 months old (black), NoTg female at 5 months old (pink), 3xTg-AD female at 5 months old (green), NoTg male at 3 months old (purple), 3xTg-AD male at 3 months old (red), NoTg male at 5 months old (yellow), 3xTg-AD male at 3 months old (blue). The data is comparing by sex NoTg and 3xTg; NoTg female and male mice at 3 months **(A)**, NoTg female and male mice at 5 months **(B)**, 3xTg female and male mice at 3 months **(C)**, and 3xTg female and male mice at 5 months **(D)**. The three-dimensional scatter plots were generated using principal coordinates analyses (PCoA) in three different axes which shows the percentage of total differences. There is significant difference on **(A)** according to ANOSIM similarity test (*R* = 0.225, *p* = 0.002), but no for (**B**, *R* = 0.082, *p* = 0.102), (**C**, *R* = 0.037, *p* = 0.231), and (**D**, *R* = 0.029, *p* = 0.275).

The analysis of the significance of differences in the beta diversities by the ANOSIM similarity test was grouping genotype (Figure 8; NoTg female vs. 3xTg female at 3 months, Figure 8A; NoTg female vs. 3xTg female at 5 months, Figure 8B; NoTg male vs. 3xTg male at 3 months, Figure 8C; NoTg male vs. 3xTg male at 5 months, Figure 8D), age (Figure 9; NoTg female 3 months vs. 5 months, Figure 9A; 3xTg female 3 months vs. 5 months, Figure 9B; NoTg male 3 months vs. 5 months, Figure 9C; 3xTg male 3 months vs. 5 months, Figure 9D), and sex (Figure 10; NoTg female vs. NoTg male at 3 months, Figure 10A; NoTg female vs. NoTg male at 5 months, Figure 10B; 3xTg female vs. 3xTg male at 3 months, Figure 10C; 3xTg female vs. 3xTg male at 5 months, Figure 10D). The main differences were observed comparing by genotypes, showing differences for all comparisons: NoTg vs. 3xTg-AD females at 3 months old (*R* = 0.184, *p* = 0.018), NoTg vs. 3xTg-AD females at 5 months old (*R* = 0.124, *p* = 0.028), NoTg vs. 3xTg-AD males at 3 months old (*R* = 0.422, *p* = 0.001), and NoTg vs. 3xTg-AD male at 5 months old (*R* = 0.174, *p* = 0.022) (Figure 8); however, the comparison by age showed only differences for NoTg male mice 3 vs. 5 months old (*R* = 0.425, *p* = 0.002) (Figure 9). Finally, when comparing by sex, there was only a difference between NoTg female vs. NoTg male at 3 months old (*R* = 0.225, *p* = 0.002) (Figure 10).

### The Genotype, Age, and Sex Are Associated With Changes in the Abundance of Bacteria Related to Neurodegenerative Diseases

**FIGURE 11:**
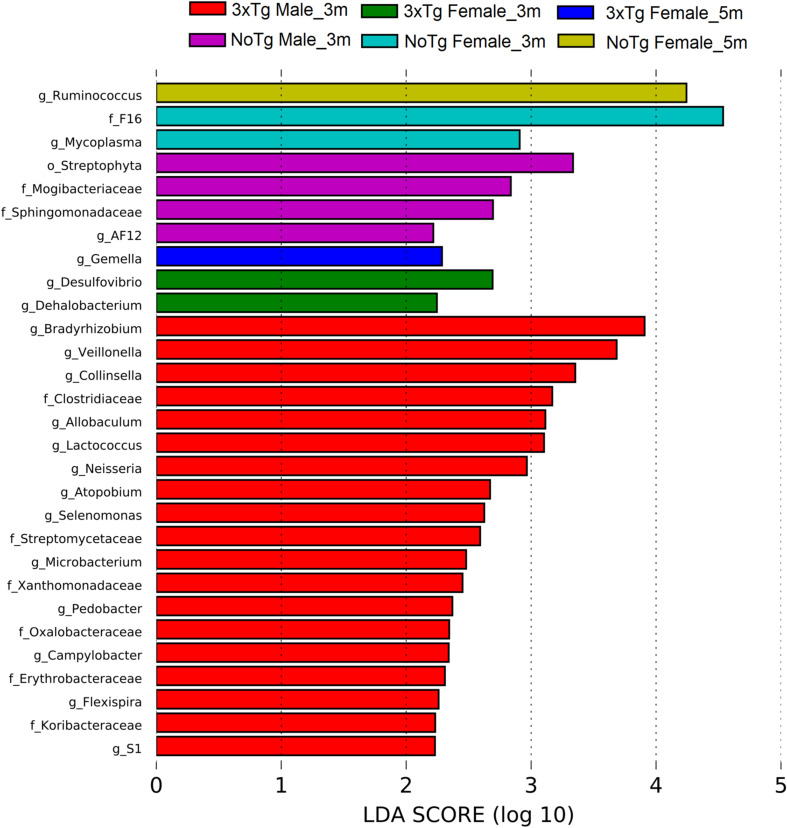
Bacteria with a statistically significant change between non-transgenic (NoTg) and Alzheimer’s disease triple-transgenic (3xTg-AD) mice at 3 and 5 months (m) old in male and female in fecal samples. The bacteria with statistical relevance are shown by colors: red color indicates 3xTg-AD male at 3 months old; green, 3xTg-AD female at 3 months old; blue, 3xTg-AD female at 5 months old; pink, NoTg male at 3 months old; cyan, NoTg female at 3 months old; light olive, NoTg female at 5 months old. Horizontal bars represent the effect size for each taxon. The length of the bars represents the *log*_10_ transformed linear discriminant analysis (LDA) score, indicated by vertical dotted lines. The threshold on the logarithmic LDA score for discriminative features was set to 2.0. The name of bacteria with a statistically significant change in the relative abundance is written alongside the horizontal lines. Taxa names are abbreviated as “o,” order; “f,” family, and “g,” genus. See Supplementary Table 5 for full taxon description and LDA score and *p* values.

The LEfSe analyses revealed bacteria with statistically significant changes in the abundance among all groups. At 3 months old, NoTg female mice had an increased abundance of members of the family F16 (phylum TM7) and the genus *Mycoplasma* (phylum Tenericutes); the 3xTg-AD female had an increase of genus *Dehalobacterium*, (phylum Firmicutes) and the genus *Desulfovibrio* (phylum Proteobacteria). At 5 months old, NoTg female had an increase in *Ruminococcus* (phylum Firmicutes), while 3xTg-AD females had an increase in *Gemella* (phylum Firmicutes). At 3 months old, NoTg male mice had an increase of the genus *AF12* (phylum Bacteroidetes), members of the family Mogibacteriaceae (phylum Firmicutes), and the family Sphingomonadaceae (phylum Proteobacteria). The 3xTg-AD male mice at 3 months old increased the family Koribacteraceae (phylum Acidobacteria); the family Streptomycetaceae; and the genera *Atopobium*, *Collinsella*, and *Microbacterium* (phylum Actinobacteria). There was also an increase in the genus *Pedobacter* (phylum Bacteroidetes); the family Clostridiaceae; genera *Allobaculum*, *Lactococcus*, *Selenomonas*, and *Veillonella* (the phylum Firmicutes); the families Erythrobacteraceae, Oxalobacteraceae, Xanthomonadaceae; and the genera *Bradyrhizobium*, *Campylobacter*, *Flexispira*, and *Neisseria* (phylum Proteobacteria); and the genus *S1* (phylum Thermotogae) (Figure 11 and Supplementary Table 5).

**FIGURE 12:**
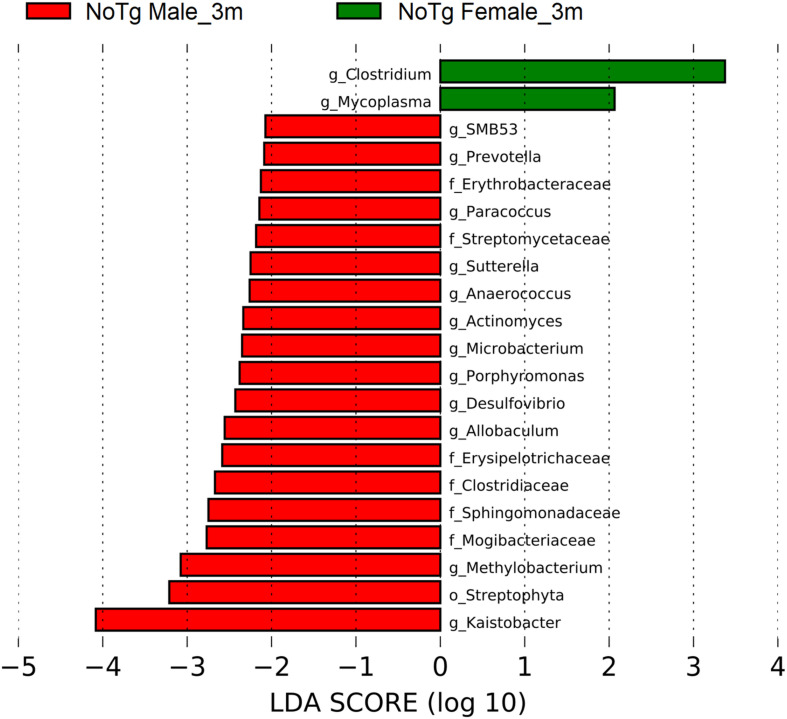
Bacteria with statistically significant change on non-transgenic (NoTg) mice at 3 and 5 months (m) old for male and female in fecal samples. The bacteria with statistical relevance are shown by colors: red color indicates NoTg male at 3 months old; green, NoTg female at 3 months old. Horizontal bars represent the effect size for each taxon. The length of the bars represents the *log*_10_ transformed linear discriminant analysis (LDA) score, indicated by vertical dotted lines. The threshold on the logarithmic LDA score for discriminative features was set to 2.0. The name of bacteria with a statistically significant change in the relative abundance is written alongside the horizontal lines. Taxa names are abbreviated as “c,” class; “o,” order; “f,” family, and “g,” genus. See Supplementary Table 6 for full taxon description and LDA score and *p* values.

A different profile in the abundances was observed at 3 months old only for NoTg mice. NoTg female had an increase in the abundance of members of the genera *Clostridium* (phylum Firmicutes) and *Mycoplasma* (phylum Tenericutes). NoTg male had an increase in the family Streptomycetaceae; genera *Actinomyces* and *Microbacterium* (phylum Actinobacteria); *Porphyromonas* and *Prevotella* (phylum Bacteroidetes); the families Clostridiaceae, Erysipelotrichaceae, Mogibacteriaceae; the genera *Allobaculum*, *Anaerococcus*, and *SMB53* (phylum Firmicutes); the families Erythrobacteraceae and Sphingomonadaceae; and the genera *Desulfovibrio*, *Kaistobacter*, *Methylobacterium*, *Paracoccus*, and *Sutterella* (phylum Proteobacteria) (Figure 12 and Supplementary Table 6).

**FIGURE 13:**
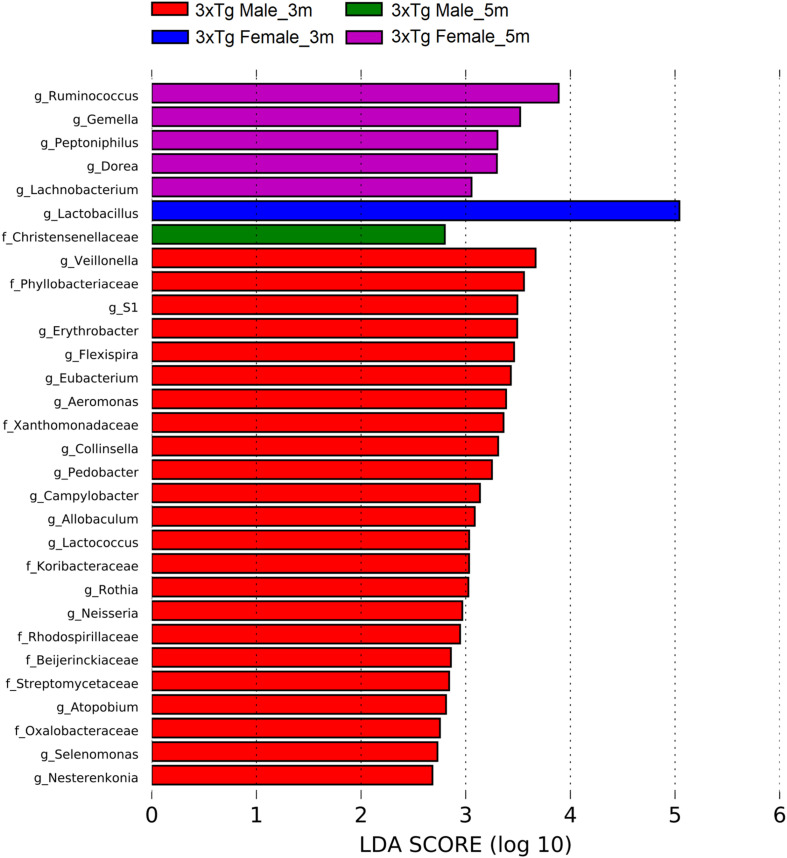
Bacteria with statistically significant change on Alzheimer’s disease triple-transgenic (3xTg-AD) mice at 3 and 5 months (m) old in male and female in fecal samples. The bacteria with statistical relevance are shown by colors: red indicates 3xTg-AD male at 3 months old, green indicates 3xTg-AD male at 5 months old, blue indicates 3xTg-AD female at 3 months old, and pink indicates 3xTg-AD female at 5 months old. Horizontal bars represent the effect size for each taxon. The length of the bars represents the log10 transformed linear discriminant analysis (LDA) score, indicated by vertical dotted lines. The threshold on the logarithmic LDA score for discriminative features was set to 2.0. The name of bacteria with a statistically significant change in the relative abundance is written alongside the horizontal lines. Taxa names are abbreviated as “f,” family, and “g,” genus. See Supplementary Table 7 for full taxon description and LDA score and *p* values.

For the transgenic mice, at 3 months old, the abundances in the 3xTg-AD female showed an increase in the genus *Lactobacillus* (phylum Firmicutes), while at 5 months old, it showed an increase in the genera *Dorea*, *Gemella*, *Lachnobacterium*, *Peptoniphilus*, and *Ruminococcus* (phylum Firmicutes). The 3xTg-AD male mice at 3 months old showed an increase in the family Koribacteraceae (phylum Acidobacteria); the family Streptomycetaceae; the genera *Atopobium*, *Collinsella*, *Nesterenkonia*, and *Rothia* (phylum Actinobacteria); the genus *Pedobacter* (phylum Bacteroidetes); the genera *Allobaculum*, *Eubacterium*, *Lactococcus*, *Selenomonas*, and *Veillonella* (phylum Firmicutes); the families Beijerinckiaceae, Oxalobacteraceae, Phyllobacteriaceae, Rhodospirillaceae, Xanthomonadaceae; the genera *Aeromonas*, *Campylobacter*, *Erythrobacter*, *Flexispira*, and *Neisseria* (phylum Proteobacteria); and the genus *S1* (phylum Thermotogae). The 3xTg-AD male at 5 months old showed an increase in the family Christensenellaceae (phylum Firmicutes) (Figure 13 and Supplementary Table 7).

## Discussion

We studied changes in the fecal microbiota associated with cognitive impairment in behavioral paradigm tests at an early adulthood of the AD transgenic mouse model. We found an impairment of memory retention of NOL in female and male 3xTg-AD mice at 5 months old, without motor activity alteration, demonstrating that such deficit in memory performance was cognitive specific. Furthermore, we observed an increase in the area occupied for β-amyloid peptide in the *subiculum* and CA1 of the dorsal hippocampus in female and male 3xTg-AD mice. Importantly, we found gut microbiota dysbiosis in female and male 3xTg-AD mice already at 3 and 5 months old, which until now are both considered within a pre-symptomatic stage of this pathology (Clark et al., 2015).

Two variants of object recognition paradigms have been reported: NOL and NOR (Denninger et al., 2018). In NOL, an object in a familiar localization is changed to a novel localization in the arena, which allows to evaluate spatial memory dependent on the dorsal hippocampus (Winters et al., 2008; Burke et al., 2012). For NOR, a familiar object is replaced by a novel object, which allows to evaluate recognition memory dependent on the perirhinal cortex (Burke et al., 2012). It is important to remark that as β-amyloid peptide accumulates in the subiculum and hippocampus in the early stage of AD (Clark et al., 2015), NOL is considered an appropriate measurement to evaluate early pathological symptomatology. Our results show that NOL learning was not affected in female and male from NoTg and 3xTg-AD genotypes. However, importantly, both sexes of 3xTg-AD mice showed a deficit in the NOL retention test at a pre-symptomatic stage of AD. These data appear to contradict the results reported by Guzmán-Ramos et al. (2012), in which they found that 3xTg-AD mice show a good performance in NOR at 5 months old; thus, the authors concluded that these mice do not show memory alterations at an early age. We consider that such a contradictory result is probably related to the fact that NOR is not a pure spatial task and therefore might not be affected by Aβ accumulation within the hippocampus. In the present study, we observed decreased exploration time of the two familiar-localization objects during NOL training session in female and male 3xTg-AD mice in comparison to NoTg mice. This result could be explained partially by impaired spatial memory information storage in 3xTg-AD mice. Previous works reported a relationship between the amount of exploration time of two familiar objects and the difference of score based on the difference in the familiar and novel object in the retention test of NOR and NOL (Albasser et al., 2009; Bello-Medina et al., 2013). Other factors suggested to impair memory consolidation of NOL task in 3xTg-AD mice are as follows: (a) low release of dopamine, norepinephrine, and glutamate (Guzmán-Ramos et al., 2012); (b) β-amyloid peptide accumulation (Clark et al., 2015); (c) tau phosphorylation related to changes in hippocampal theta oscillations and decrease in excitability in hippocampal neurons (Siddhartha et al., 2018); and (d) low dendritic spine density (Zou et al., 2015; Kommaddi et al., 2018). Importantly, in such previous studies that have not observed cognitive decline, they do report histopathological and synaptic plasticity alterations that could interfere with NOL consolidation memory. On the other hand, Billings et al. (2005) found earlier cognitive impairment already manifested at 4 months old, with deficits in long-term retention in Morris water maze, inhibitory avoidance task, as well as with concomitant accumulation of intraneuronal β-amyloid peptide in the hippocampus and amygdala. This work agrees with our results, where we found a NOL impairment in female and male 3xTg-AD at 5 months old that could be related to the β-amyloid accumulation observed at 5 months old and to gut microbiota dysbiosis already observed at 3 months and confirmed at 5 months old. In sum, our results strongly suggest that for 3xTg-AD mice, 5 months old cannot be any longer considered a pre-symptomatic stage of AD; we propose instead that this age should be conceptualized as an early symptomatic stage of AD.

Epidemiological and experimental findings suggest that AD has higher prevalence and incidence and symptoms are stronger in females than males in AD human patients as well as mouse models (Bachman et al., 1992; Ruitenberg et al., 2001; Carroll et al., 2010a; Creighton et al., 2019). In our results, we did not find any significant sex-related differences in motor activity, learning, NOL memory retention, or β-amyloid accumulation in the *subiculum* and CA1 in 3xTg-AD mice at the early symptomatic stage (5 months old). This lack of sex-related differences could be explained by previous reports that show that β-amyloid peptide levels did not differ between female and male until 9 months (Hirata-Fukae et al., 2008). Sex-related differences appear, however, at the symptomatic stage of AD due to loss of ovarian steroids related to menopause women aging (Carroll et al., 2007, 2010b; Carroll and Pike, 2008). Concomitantly, female 3xTg-AD mice at 5 months old are too young for showing estrogens and progesterone reduction. Furthermore, motor activities such as speed, traveled distance, and resting time alteration in female and male 3xTg-AD mice were similar. Previous studies have demonstrated that motor activity alteration occurs in the symptomatic stage of AD in female and male 3xTg-AD mice at 6 and 16 months old (Stover et al., 2015; Garvock-de Montbrun et al., 2019).

We characterized gut microbiota and evaluated its association with the trigger and development of AD. Our results documented that aging induces gut microbiota alterations; as male NoTg of 5 months old has an increase in Firmicutes and Actinobacteria and a decrease in Proteobacteria, Fusobacteria, Cyanobacteria, and Bacteroidetes phyla in comparison with male NoTg of 3 months old. A previous report indicates that Firmicutes, Proteobacteria, and Bacteroidetes are dominant phyla in APP^swe^/PS1^Δ^E9 mice at 1 and 3 months old (Zhang et al., 2017). The decrease of Actinobacteria, Proteobacteria, Fusobacteria, and Cyanobacteria relative abundance has been reported as an indicator of aging in humans (Mariat et al., 2009) and male mice (Zhang et al., 2013) but not in female mice (Langille et al., 2014), as observed in our results. However, in female 3xTg-AD of 5 months old, a decrease in Actinobacteria phylum was observed in comparison with 3xTg-AD of 3 months old. This change could be related to the progression of AD due to that this decrease was not observed in NoTg mice of 3 and 5 months old. It is important to notice that the phylum Actinobacteria has been related to beneficial health effects like anti-inflammatory properties and decreased intestinal permeability (Vogt et al., 2017). Interestingly, the 3xTg-AD males have no differences between 3 and 5 months, which could be a sign of early aging even at an early age.

Furthermore, we observed a decrease in the relative abundance of TM7 in female and male 3xTg-AD mice at 3 and 5 months old in comparison to NoTg mice, while an increase was found in the relative abundance of Fusobacteria and Cyanobacteria in female and male 3xTg-AD mice at 5 months old in comparison to NoTg mice. This effect has been also reported in P301L mice, a tauopathy model at 3 and 6 months old (Sun et al., 2019), and in AD patients (Vogt et al., 2017). Also, a decrease in relative abundance in both TM7 and Cyanobacteria has been documented in female 3xTg-AD mice at 9 months old (Syeda et al., 2018). This evidence agrees with our results and suggests that 3xTg-AD mice at 3 and 5 months old may have similar alterations to that observed in the previously described symptomatic stage of AD.

Diversity in gut microbiota is an indicator of richness and abundance of species of microorganisms present in a sample. We analyzed Shannon and Simpson indexes and found significant differences between male and female NoTg mice at 3 and 5 months old. These results suggest that bacterial diversity in the mice hybrid strain B6129SF1/J (NoTg) changes concerning sex. This agrees with other studies that show sexual differences in microbiota in other mouse strains (Org et al., 2016; Kozik et al., 2017; Elderman et al., 2018). However, until now, there are no reports that compare alpha diversity related to sex in the mouse hybrid strain B6129SF1/J used as the genetic background for the 3xTg-AD mice. It is important to note that, in our study, there are no differences between genotypes at any of the two ages analyzed. This could be because in such pre-symptomatic age of AD is too early to observe these subtle alterations. Our results agree with those reported by Bonfili et al. (2017), where they show no differences in alpha diversity in 3xTg-AD mice at 2, 3, 4.5, and 6 months old. This same effect was observed in P301L tau transgenic mice at 1, 3, and 6 months old (Sun et al., 2019). However, it has been demonstrated that 9 months old 3xTg-AD mice showed lower alpha diversity than NoTg mice (Syeda et al., 2018). This suggests that alpha diversity may not be a meaningful indicator of the development of AD at early or pre-symptomatic stages.

Now, regarding the beta diversity of gut microbiota, we found differences in female and male 3xTg-AD mice in comparison to NoTg mice at 3 and 5 months old. Male NoTg mice at 3 months old exhibited a statistically significant difference regarding 5 months old. The same effects were observed in 3xTg-AD mice at 2, 3, 4.5, and 6 months old (Bonfili et al., 2017) and at 9 months old (Syeda et al., 2018), including APPPS1 mice at 8 months old (Harach et al., 2017). These results suggest that beta diversity changes might be associated with the triggering and progression of AD. On the other hand, we found that beta diversity in female NoTg is different from male NoTg at 3 months old. This result was observed in a different strain of mice such as C57BL/6J, C3H/HeJ, and DBA/2J; this effect is dependent on sex hormone development during puberty and early adulthood; if female mice are gonadectomized, the gut microbiota change. This alteration was prevented by hormonal substitution (Org et al., 2016).

The present study contributes to the characterization of gut microbiota in NoTg and 3xTg-AD mice related to age and sex in the early symptomatic stage of AD. It is important to mention that a decrease in Actinobacteria phylum observed in 3xTg-AD mice is related to the triggering and progression of AD. *Bifidobacterium* is a principal bacterium of Actinobacteria phylum that participates in intestinal homeostasis, and the gut–brain axis modulates the GABAergic system in the intestine (Binda et al., 2018). A decrease in Actinobacteria contributes to tau pathogenesis and cognitive decline (Sun et al., 2019). This loss in the relative abundance of Actinobacteria could be related to the memory retention deficit of NOL observed in this study. The TM7 phylum has been identified in a variety of natural habitats such as the skin, genital tract, and gastrointestinal tract. TM7 induced upregulation of many genes for biosynthesis of essential amino acids such as histidine, isoleucine, leucine, lysine, methionine, phenylalanine, threonine, tryptophan, and valine (He et al., 2015). Tryptophan, tyrosine, and phenylalanine are biosynthetic precursors for the neurotransmitters such as serotonin, dopamine, and norepinephrine (Fernstrom, 1994). A decrease in TM7 phylum could alter neuronal communication in 3xTg-AD mice, which affects information processing in memory retention of NOL.

Concerning the results of the LEfSe analysis in female and male NoTg at 3 and 5 months old in fecal samples, we found bacteria with a statistically significant change in LDA scores such as F16 (phylum TM7), genus *Mycoplasma* (phylum Tenericutes), Ruminococcus (phylum Firmicutes), AF12 (phylum Bacteroidetes), family Mogibacteriaceae (phylum Firmicutes), family *Sphingomonadaceae* (phylum Proteobacteria), genera *Clostridium* (phylum Firmicutes), *Mycoplasma* (phylum Tenericutes), family Streptomycetaceae, genera *Actinomyces* and *Microbacterium* (phylum Actinobacteria), *Porphyromonas* and *Prevotella* (phylum Bacteroidetes), families Clostridiaceae, Erysipelotrichaceae, Mogibacteriaceae, genera *Allobaculum*, *Anaerococcus*, *SMB53* (phylum Firmicutes), families Erythrobacteraceae, Sphingomonadaceae, genera *Desulfovibrio*, *Kaistobacter*, *Methylobacterium*, *Paracoccus*, and *Sutterella* (phylum Proteobacteria). These bacteria were found in the healthy gut in NoTg mice, maintaining homeostasis of microbiota with normal changes associated with aging and sex hormones dynamics (Zhang et al., 2013; Org et al., 2016). Furthermore, the results of the LEfSe analysis in female and male 3xTg-AD mice at 3 and 5 months old in fecal samples, show bacteria with a statistically significant change in LDA scores such as *Xanthomonadaceae*, *Oxalobacteraceae*, *Streptomycetaceae*, *Koribacteraceae*, and *Streptomycetaceae* families, *Gemella*, *Dehalobacterium*, *Clostridium*, *Allobaculum*, *Selenomonas*, *Veillonella*, *Lactococcus*, *Desulfovibrio*, *Bradyrhizobium*, *Campylobacter*, *Erythrobacter*, *Neisseria*, *Flexispira*, *Microbacterium*, *Collinsella*, *Atopobium*, *Pedobacter*, and the S1 genera. These microorganisms have been associated with both pre-clinical models and patients who present AD mainly; however, other bacteria are related to aging, cognitive decline, cerebral damage, and inflammatory response in mice and humans (Thomas et al., 2012; Wang et al., 2016; Bonfili et al., 2017; Harach et al., 2017; Morris et al., 2017; Vogt et al., 2017; Zhang et al., 2017; Aguayo et al., 2018; Alonso et al., 2018; Antonets et al., 2018; Bäuerl et al., 2018; Dong et al., 2018; Zhuang et al., 2018; Haran et al., 2019; Li et al., 2019; Zhan et al., 2019; Beydoun et al., 2020; Na et al., 2020; Westfall et al., 2020). These results suggest that bacteria families and genera are representative microorganisms of gut microbiota of disease that could be considered a useful tool for diagnostic as well as a progression biomarker of AD.

Finally, it is important to mention some limitations of this study. Although some bacteria modifications have been reported in human AD patients, this, as other pre-clinical results, must be interpreted with caution because the results of this mouse transgenic model may not directly translate to the humans. Additionally, it is necessary to study the consequences and causalities when manipulating microbiota in the 3xTg-AD model. In this regard, it would be interesting to modulate gut microbiota during the first 5 months of age in 3xTg-AD and then evaluate the cognitive impairment associated with this neurodegenerative pathology in such early symptomatic stage of AD.

## Conclusion

Our results suggest that 5 months old 3xTg-AD mice cannot be considered a the pre-symptomatic stage of AD. We propose that this age should be considered the early symptomatic stage of AD. Additionally, we found gut microbiota alterations at 3 months old in 3xTg-AD mice that could be considered and used as early biomarkers for the diagnostic and progression of AD. These biomarkers are Actinobacteria and TM7 phylum alterations as well as beta diversity significant changes and an increase in specific bacteria families and genera, i.e., *Gemella*, *Allobacullum*, and *Selenomonas*.

## Data Availability Statement

The corresponding FASTQ sequence files for all samples used in this study were deposited in the NCBI BioProject repository (Accession Number: PRJNA648144) link: https://www.ncbi.nlm.nih.gov/bioproject/PRJNA648144.

## Ethics Statement

The animal study was reviewed and approved by Comité de Bioética del Instituto de Neurobiología, Universidad Nacional Autónoma de México.

## Author Contributions

PB-M, JG-M, SD-C, and GP-L designed the research. PB-M and FH-Q performed the research. PB-M, FH-Q, and JG-M analyzed the data. PB-M, FH-Q, JG-M, SD-C, and GP-L wrote the manuscript. PB-M, FH-Q, MP-M, DG-F, GC-P, and GP-L revised the manuscript. All authors contributed to the article and approved the submitted version.

## Conflict of Interest

The authors declare that the research was conducted in the absence of any commercial or financial relationships that could be construed as a potential conflict of interest.
