# Supplementary material for: Spatial Memory and Gut Microbiota Alterations Are Already Present in Early Adulthood in a Pre-clinical Transgenic Model of Alzheimer’s Disease
Source: Front Neurosci. 2021 Apr 29;15:595583. doi: 10.3389/fnins.2021.595583 (PMC8116633; doi:10.3389/fnins.2021.595583)
Supplement: Supplementary file 1 [file Data_Sheet_1.zip › Table 1.DOCX]

| **Supplementary Table S1**  Relative abundance of fecal samples from NoTg and 3xTg mice. | | | |
| --- | --- | --- | --- |
| Bacteria | 3 months old | 5 months old | *p*- value |
| Female NoTg | | | |
| Firmicutes | 79.06 ± 12.99 | 87.51 ± 6.78 | 0.247 |
| Bacteroidetes | 9.78 ± 8.04 | 4.20 ± 3.79 | 0.059 |
| Actinobacteria | 1.01 ± 0.95 | 0.85 ± 1.31 | 0.364 |
| Proteobacteria | 1.49 ± 2.07 | 0.45 ± 0.73 | 0.597 |
| TM7 | 8.24 ± 6.84 | 6.69 ± 4.94 | 0.762 |
| Fusobacteria | 0.00 ± 0.00 | 0.00 ± 0.00 | 0.147 |
| Cyanobacteria | 0.35 ± 0.36 | 0.26 ± 0.25 | 0.257 |
| Female 3xTg | | | |
| Firmicutes | 86.82 ± 8.50 | 91.28 ± 3.86 | 0.226 |
| Bacteroidetes | 9.64 ± 7.73 | 5.93 ± 2.79 | 0.199 |
| Actinobacteria | 0.68 ± 0.52 | 0.29 ± 0.31 | **0.049** |
| Proteobacteria | 0.83 ± 0.91 | 0.49 ± 0.66 | 0.290 |
| TM7 | 1.81 ± 1.95 | 1.79 ± 2.27 | 0.880 |
| Fusobacteria | 0.00 ± 0.00 | 0.01 ± 0.02 | 0.456 |
| Cyanobacteria | 0.19 ± 0.17 | 0.13 ± 0.13 | 0.364 |
| Male NoTg | | | |
| Firmicutes | 79.91 ± 8.43 | 88.50 ± 6.32 | **0.017** |
| Bacteroidetes | 10.89 ± 5.83 | 4.53 ± 4.11 | **0.008** |
| Actinobacteria | 1.46 ± 1.13 | 0.55 ± 1.37 | **0.005** |
| Proteobacteria | 2.40 ± 2.86 | 0.47 ± 0.88 | **0.004** |
| TM7 | 4.70 ± 3.22 | 5.80 ± 5.13 | 0.718 |
| Fusobacteria | 0.01 ± 0.01 | 0.00 ± 0.00 | **0.011** |
| Cyanobacteria | 0.62 ± 0.40 | 0.14 ± 0.15 | **0.001** |
| Male 3xTg | | | |
| Firmicutes | 82.84 ± 15.99 | 88.98 ± 6.51 | 0.624 |
| Bacteroidetes | 7.44 ± 5.52 | 8.32 ± 5.45 | 0.744 |
| Actinobacteria | 4.26 ± 5.75 | 0.49 ± 0.63 | 0.072 |
| Proteobacteria | 3.37 ± 7.79 | 0.25 ± 0.25 | 0.806 |
| TM7 | 1.20 ± 0.91 | 1.63 ± 0.93 | 0.253 |
| Fusobacteria | 0.65 ± 1.23 | 0.00 ± 0.00 | 0.235 |
| Cyanobacteria | 0.21 ± 0.20 | 0.16 ± 0.17 | 0.604 |
| Data shows the Mean ± standard deviation as indicated (see Fig. 4). The *p*-values were calculated using Mann-Whitney U test comparing data of 3 vs. 5 months old. *p* < 0.05 are considered statistically significant and are marked in bold font. | | | |
