# Supplementary material for: Spatial Memory and Gut Microbiota Alterations Are Already Present in Early Adulthood in a Pre-clinical Transgenic Model of Alzheimer’s Disease
Source: Front Neurosci. 2021 Apr 29;15:595583. doi: 10.3389/fnins.2021.595583 (PMC8116633; doi:10.3389/fnins.2021.595583)
Supplement: Supplementary file 1 [file Data_Sheet_1.zip › Table 2.DOCX]

| **Supplementary Table S2**  Statistical tests for comparison of means of phyla from NoTg and 3xTg mice. | | | | | | | |
| --- | --- | --- | --- | --- | --- | --- | --- |
| Groups | Firmicutes | Bacteroidetes | Actinobacteria | Proteobacteria | TM7 | Fusobacteria | Cyanobacteria |
| NoTg vs. 3xTg F-3 m | 0.082 | 0.705 | 0.597 | 0.406 | **0.002** | 0.550 | 0.880 |
| NoTg vs. 3xTg F-5 m | 0.257 | 0.226 | 0.940 | 0.226 | **0.007** | 0.909 | 0.806 |
| NoTg vs. 3xTg M-3 m | 0.205 | 0.181 | 0.944 | 0.139 | **0.003** | **0.003** | **0.049** |
| NoTg vs. 3xTg M-5 m | 0.970 | 0.102 | 0.063 | 0.732 | **0.007** | 0.563 | 0.260 |
| NoTg F vs. NoTg M, 3 m | 0.944 | 0.526 | 0.260 | 0.078 | 0.231 | 0.941 | **0.007** |
| NoTg F vs. NoTg M, 5 m | 0.673 | 0.833 | 0.572 | 0.944 | 0.573 | 0.501 | 0.849 |
| 3xTg F vs. 3xTg M, 3 m | 0.880 | 0.650 | 0.290 | 0.364 | 0.650 | **0.031** | 0.082 |
| 3xTg F vs. 3xTg M, 5 m | 0.514 | 0.369 | 0.624 | 0.191 | 0.514 | 0.829 | 0.226 |
| Data shows *p* values comparing de data as indicated (see Fig. 4). The *p*-values were calculated using Mann-Whitney U test. *p* < 0.05 are considered statistically significant and are marked in bold font. Abbreviations: F, female; M, Male; m, months. | | | | | | | |
