# Supplementary material for: Spatial Memory and Gut Microbiota Alterations Are Already Present in Early Adulthood in a Pre-clinical Transgenic Model of Alzheimer’s Disease
Source: Front Neurosci. 2021 Apr 29;15:595583. doi: 10.3389/fnins.2021.595583 (PMC8116633; doi:10.3389/fnins.2021.595583)
Supplement: Supplementary file 1 [file Data_Sheet_1.zip › Table 3.DOCX]

| **Supplementary Table S3**  Diversity indexes of fecal samples from NoTg and 3xTg mice. | | | |
| --- | --- | --- | --- |
| Index | 3 months old | 5 months old | *p*- value |
| Female NoTg | | | |
| ^a^Observed | 594.50 ± 256.87 | 507.20 ± 277.29 | 0.475 |
| ^a^Chao1 index | 924.10 ± 354.11 | 816.00 ± 409.55 | 0.536 |
| ^b^Shannon index | 3.78 ± 0.67 | 3.55 ± 0.53 | 0.364 |
| ^b^Simpson index | 0.92 ± 0.07 | 0.91 ± 0.05 | 0.496 |
| Female 3xTg | | | |
| ^a^Observed | 614.30 ± 294.40 | 546.00 ± 215.62 | 0.561 |
| ^a^Chao1 index | 976.13 ± 426.11 | 903.96 ± 358.24 | 0.687 |
| ^b^Shannon index | 3.85 ± 0.49 | 3.77 ± 0.78 | 0.880 |
| ^b^Simpson index | 0.92 ± 0.04 | 0.88 ± 0.13 | 0.597 |
| Male NoTg | | | |
| ^a^Observed | 702.18 ± 146.87 | 615.82 ± 221.54 | 0.294 |
| ^a^Chao1 index | 1055.31 ± 219.18 | 1018.65 ± 344.21 | 0.769 |
| ^b^Shannon index | 4.30 ± 0.22 | 3.98 ± 0.54 | 0.053 |
| ^b^Simpson index | 0.96 ± 0.01 | 0.94 ± 0.05 | 0.250 |
| Male 3xTg | | | |
| ^a^Observed | 618.40 ± 385.80 | 604.33 ± 247.36 | 0.927 |
| ^a^Chao1 index | 1003.45 ± 621.57 | 960.57 ± 370.08 | 0.859 |
| ^b^Shannon index | 4.00 ± 0.75 | 4.02 ± 0.30 | 0.744 |
| ^b^Simpson index | 0.93 ± 0.06 | 0.94 ± 0.02 | 0.683 |
| Data shows the Mean ± standard deviation as indicated (see Figs. 5 and 6). The *p*-values were calculated using T test^a^ or Mann-Whitney U test^b^ comparing data of 3 vs. 5 months old. *p* < 0.05 are considered statistically significant. | | | |
