# Supplementary material for: Spatial Memory and Gut Microbiota Alterations Are Already Present in Early Adulthood in a Pre-clinical Transgenic Model of Alzheimer’s Disease
Source: Front Neurosci. 2021 Apr 29;15:595583. doi: 10.3389/fnins.2021.595583 (PMC8116633; doi:10.3389/fnins.2021.595583)
Supplement: Supplementary file 1 [file Data_Sheet_1.zip › Table 4.DOCX]

| **Supplementary Table S4**  Statistical tests for comparison of means of alpha diversity indexes from NoTg and 3xTg mice. | | | | |
| --- | --- | --- | --- | --- |
| Groups | Observed^a^ | Chao1^a^ | Shannon^b^ | Simpson^b^ |
| NoTg vs 3xTg F-3 m | 0.874 | 0.770 | 0.940 | 0.705 |
| NoTg vs 3xTg F-5 m | 0.731 | 0.615 | 0.290 | 0.650 |
| NoTg vs 3xTg M-3 m | 0.511 | 0.798 | 0.360 | 0.205 |
| NoTg vs 3xTg M-5 m | 0.914 | 0.721 | 0.518 | 0.184 |
| NoTg female vs NoTg M-3 m | 0.247 | 0.315 | **0.041** | **0.035** |
| NoTg female vs NoTg M-5 m | 0.332 | 0.233 | **0.041** | **0.035** |
| 3xTg female vs 3xTg M-3 m | 0.979 | 0.910 | 0.450 | 0.597 |
| 3xTg female vs 3xTg M-5 m | 0.590 | 0.739 | 0.683 | 0.568 |
| Data shows *p* values comparing de data as indicated (see Figs. 5 and 6). The *p*-values were calculated using T test^a^ or Mann-Whitney U test ^b^. *p* values <0.05 are considered statistically significant and are marked in bold font. Abbreviations: F, female; M, Male; m, months. | | | | |
