# Supplementary material for: Spatial Memory and Gut Microbiota Alterations Are Already Present in Early Adulthood in a Pre-clinical Transgenic Model of Alzheimer’s Disease
Source: Front Neurosci. 2021 Apr 29;15:595583. doi: 10.3389/fnins.2021.595583 (PMC8116633; doi:10.3389/fnins.2021.595583)
Supplement: Supplementary file 1 [file Data_Sheet_1.zip › Table 5.DOCX]

| **Supplementary Table S5** | | |
| --- | --- | --- |
| Taxa identified in fecal samples from NoTg and 3xTg mice after LEfSe analysis. | | |
| **Bacteria** | **LDA** | ***p*- value** |
| NoTg Female 3 months old | | |
| p_TM7; c_TM73; o_CW040; f_F16 | 4.54 | **0.00004** |
| p_Tenericutes; c_Mollicutes; o_Mycoplasmatales; f_Mycoplasmataceae; g_Mycoplasma | 2.91 | **0.00305** |
| 3xTg Female 3 months old | | |
| p_Proteobacteria; c_Deltaproteobacteria; o_Desulfovibrionales; f_Desulfovibrionaceae; g_Desulfovibrio | 2.69 | **0.00108** |
| p_Firmicutes; c_Clostridia; o_Clostridiales; f_Dehalobacteriaceae; g_Dehalobacterium | 2.25 | **0.00214** |
| NoTg Female 5 months old | | |
| p_Firmicutes; c_Clostridia; o_Clostridiales; f_Ruminococcaceae; g_Ruminococcus | 4.24 | **0.00176** |
| 3xTg Female 5 months old | | |
| p_Firmicutes; c_Bacilli; o_Gemellales; f_Gemellaceae; g_Gemella | 2.29 | **0.00137** |
| NoTg Male 3 months old | | |
| p_Bacteroidetes; c_Bacteroidia; o_Bacteroidales; f_Rikenellaceae; g_AF12 | 2.22 | **0.00027** |
| p_Firmicutes; c_Clostridia; o_Clostridiales; f_Mogibacteriaceae | 2.84 | **0.00025** |
| p_Proteobacteria; c_Alphaproteobacteria; o_Sphingomonadales; f_Sphingomonadaceae | 2.70 | **0.00152** |
| p_Cyanobacteria; c_Chloroplast; o_Streptophyta | 3.34 | **0.00212** |
| 3xTg Male 3 months old | | |
| p_Proteobacteria; c_Gammaproteobacteria; o_Xanthomonadales; f_Xanthomonadaceae | 2.45 | **0.00003** |
| p_Firmicutes; c_Bacilli; o_Lactobacillales; f_Streptococcaceae; g_Lactococcus | 3.10 | **0.00002** |
| p_Actinobacteria; c_Coriobacteriia; o_Coriobacteriales; f_Coriobacteriaceae; g_Atopobium | 2.67 | **0.00011** |
| p_Firmicutes; c_Erysipelotrichi; o_Erysipelotrichales; f_Erysipelotrichaceae; g_Allobaculum | 3.11 | **0.00015** |
| p_Proteobacteria; c_Betaproteobacteria; o_Burkholderiales; f_Oxalobacteraceae | 2.34 | **0.00057** |
| p_Acidobacteria; c_Acidobacteriia; o_Acidobacteriales; f_Koribacteraceae | 2.23 | **0.00102** |
| p_Proteobacteria; c_Alphaproteobacteria; o_Sphingomonadales; f_Erythrobacteraceae | 2.31 | **0.00077** |
| p_Bacteroidetes; c_Sphingobacteriia; o_Sphingobacteriales; f_Sphingobacteriaceae; g_Pedobacter | 2.37 | **0.00102** |
| p_Firmicutes; c_Clostridia; o_Clostridiales; f_Veillonellaceae; g_Selenomonas | 2.63 | **0.00102** |
| p_Thermotogae; c_Thermotogae; o_Thermotogales; f_Thermotogaceae; g_S1 | 2.23 | **0.00137** |
| p_Proteobacteria; c_Epsilonproteobacteria; o_Campylobacterales; f_Helicobacteraceae; g_Flexispira | 2.26 | **0.00192** |
| p_Proteobacteria; c_Epsilonproteobacteria; o_Campylobacterales; f_Campylobacteraceae; g_Campylobacter | 2.34 | **0.00222** |
| p_Firmicutes; c_Clostridia; o_Clostridiales; f_Veillonellaceae; g_Veillonella | 3.68 | **0.00206** |
| p_Actinobacteria; c_Actinobacteria; o_Actinomycetales; f_Microbacteriaceae; g_Microbacterium | 2.48 | **0.00243** |
| p_Proteobacteria; c_Betaproteobacteria; o_Neisseriales; f_Neisseriaceae; g_Neisseria | 2.97 | **0.00246** |
| p_Proteobacteria; c_Alphaproteobacteria; o_Rhizobiales; f_Bradyrhizobiaceae; g_Bradyrhizobium | 3.91 | **0.00295** |
| p_Actinobacteria; c_Coriobacteriia; o_Coriobacteriales; f_Coriobacteriaceae; g_Collinsella | 3.35 | **0.00366** |
| p_Actinobacteria; c_Actinobacteria; o_Actinomycetales; f_Streptomycetaceae | 2.59 | **0.00382** |
| p_Firmicutes; c_Clostridia; o_Clostridiales; f_Clostridiaceae | 3.17 | **0.00405** |
| LDA: Linear discriminant analysis. The threshold on the logarithmic LDA score for discriminative features was set to 2.0 as indicated. “p” phylum, “c”, class; “o”, order; “f”, family; “g”, genus. The *p*-values were calculated using Kruskal-Wallis test. *p* ≤ 0.05 are considered statistically significant and are marked in bold font (see Figure 10). | | |
