# Supplementary material for: Spatial Memory and Gut Microbiota Alterations Are Already Present in Early Adulthood in a Pre-clinical Transgenic Model of Alzheimer’s Disease
Source: Front Neurosci. 2021 Apr 29;15:595583. doi: 10.3389/fnins.2021.595583 (PMC8116633; doi:10.3389/fnins.2021.595583)
Supplement: Supplementary file 1 [file Data_Sheet_1.zip › Table 6.DOCX]

| **Supplementary Table S6** | | |
| --- | --- | --- |
| Taxa identified in fecal samples from NoTg mice after LEfSe analysis. | | |
| **Bacteria** | **LDA** | ***p*- value** |
| NoTg Female 3 months old | | |
| p_Tenericutes; c_Mollicutes; o_Mycoplasmatales; f_Mycoplasmataceae; g_Mycoplasma | 2.06 | **0.02060** |
| p_Firmicutes; c_Clostridia; o_Clostridiales; f_Clostridiaceae; g_Clostridium | 3.37 | **0.03810** |
| NoTg Male 3 months old | | |
| p_Firmicutes; c_Clostridia; o_Clostridiales; f_Clostridiaceae; g_SMB53 | 2.07 | **0.04833** |
| p_Bacteroidetes; c_Bacteroidia; o_Bacteroidales; f_Paraprevotellaceae; g_Prevotella | 2.09 | **0.03515** |
| p_Proteobacteria; c_Alphaproteobacteria; o_Sphingomonadales; f_Erythrobacteraceae | 2.13 | **0.00055** |
| p_Proteobacteria; c_Alphaproteobacteria; o_Rhodobacterales; f_Rhodobacteraceae; g_Paracoccus | 2.14 | **0.00660** |
| p_Actinobacteria; c_Actinobacteria; o_Actinomycetales; f_Streptomycetaceae | 2.18 | **0.01679** |
| p_Proteobacteria; c_Betaproteobacteria; o_Burkholderiales; f_Alcaligenaceae; g_Sutterella | 2.25 | **0.01742** |
| p_Firmicutes; c_Clostridia; o_Clostridiales; f_Tissierellaceae; g_Anaerococcus | 2.26 | **0.00012** |
| p_Actinobacteria; c_Actinobacteria; o_Actinomycetales; f_Actinomycetaceae; g_Actinomyces | 2.33 | **0.03515** |
| p_Actinobacteria; c_Actinobacteria; o_Actinomycetales; f_Microbacteriaceae; g_Microbacterium | 2.35 | **0.00134** |
| p_Bacteroidetes; c_Bacteroidia; o_Bacteroidales; f_Porphyromonadaceae; g_Porphyromonas | 2.38 | **0.03515** |
| p_Proteobacteria; c_Deltaproteobacteria; o_Desulfovibrionales; f_Desulfovibrionaceae; g_Desulfovibrio | 2.43 | **0.00043** |
| p_Firmicutes; c_Erysipelotrichi; o_Erysipelotrichales; f_Erysipelotrichaceae; g_Allobaculum | 2.56 | **0.00101** |
| p_Firmicutes; c_Erysipelotrichi; o_Erysipelotrichales; f_Erysipelotrichaceae | 2.58 | **0.00516** |
| p_Firmicutes; c_Clostridia; o_Clostridiales; f_Clostridiaceae | 2.67 | **0.00138** |
| p_Proteobacteria; c_Alphaproteobacteria; o_Sphingomonadales; f_Sphingomonadaceae | 2.75 | **0.00202** |
| p_Firmicutes; c_Clostridia; o_Clostridiales; f_Mogibacteriaceae | 2.77 | **0.00007** |
| p_Proteobacteria; c_Alphaproteobacteria; o_Rhizobiales; f_Methylobacteriaceae; g_Methylobacterium | 3.08 | **0.00811** |
| p_Cyanobacteria; c_Chloroplast; o_Streptophyta | 3.21 | **0.00642** |
| p_Proteobacteria; c_Alphaproteobacteria; o_Sphingomonadales; f_Sphingomonadaceae; g_Kaistobacter | 4.08 | **0.01587** |
| LDA: Linear discriminant analysis. The threshold on the logarithmic LDA score for discriminative features was set to 2.0 as indicated. “p” phylum, “c”, class; “o”, order; “f”, family; “g”, genus. The *p*-values were calculated using Kruskal-Wallis test. *p* value ≤ 0.05 are considered statistically significant and are marked in bold font (see Figure 11). | | |
