# Supplementary material for: Spatial Memory and Gut Microbiota Alterations Are Already Present in Early Adulthood in a Pre-clinical Transgenic Model of Alzheimer’s Disease
Source: Front Neurosci. 2021 Apr 29;15:595583. doi: 10.3389/fnins.2021.595583 (PMC8116633; doi:10.3389/fnins.2021.595583)
Supplement: Supplementary file 1 [file Data_Sheet_1.zip › Table 7.DOCX]

| **Supplementary Table S7** | | |
| --- | --- | --- |
| Taxa identified in fecal samples from 3xTg mice after LEfSe analysis. | | |
| **Bacteria** | **LDA** | ***p*- value** |
| 3xTg Female 3 months old | | |
| p_Firmicutes; c_Bacilli; o_Lactobacillales; f_Lactobacillaceae; g_Lactobacillus | 5.04 | **0.0462** |
| 3xTg Female 5 months old | | |
| p_Firmicutes; c_Clostridia; o_Clostridiales; f_Lachnospiraceae; g_Dorea | 3.30 | **0.0123** |
| p_Firmicutes; c_Clostridia; o_Clostridiales; f_Tissierellaceae; g_Peptoniphilus | 3.30 | **0.0490** |
| p_Firmicutes; c_Clostridia; o_Clostridiales; f_Lachnospiraceae; g_Lachnobacterium | 3.06 | **0.0166** |
| p_Firmicutes; c_Bacilli; o_Gemellales; f_Gemellaceae; g_Gemella | 3.52 | **0.0304** |
| p_Firmicutes; c_Clostridia; o_Clostridiales; f_Ruminococcaceae; g_Ruminococcus | 3.89 | **0.0134** |
| 3xTg Male 3 months old | | |
| p_Proteobacteria; c_Epsilonproteobacteria; o_Campylobacterales; f_Campylobacteraceae; g_Campylobacter | 3.14 | **0.0124** |
| p_Firmicutes; c_Erysipelotrichi; o_Erysipelotrichales; f_Erysipelotrichaceae; g_Eubacterium | 3.43 | **0.0129** |
| p_Firmicutes; c_Erysipelotrichi; o_Erysipelotrichales; f_Erysipelotrichaceae; g_Allobaculum | 3.09 | **0.0074** |
| p_Acidobacteria; c_Acidobacteriia; o_Acidobacteriales; f_Koribacteraceae | 3.03 | **0.0170** |
| p_Firmicutes; c_Clostridia; o_Clostridiales; f_Veillonellaceae; g_Selenomonas | 2.73 | **0.0170** |
| p_Proteobacteria; c_Alphaproteobacteria; o_Sphingomonadales; f_Erythrobacteraceae; g_Erythrobacter | 3.49 | **0.0170** |
| p_Actinobacteria; c_Coriobacteriia; o_Coriobacteriales; f_Coriobacteriaceae; g_Atopobium | 2.81 | **0.0097** |
| p_Actinobacteria; c_Actinobacteria; o_Actinomycetales; f_Micrococcaceae; g_Rothia | 3.03 | **0.0129** |
| p_Firmicutes; c_Clostridia; o_Clostridiales; f_Veillonellaceae; g_Veillonella | 3.67 | **0.0238** |
| p_Actinobacteria; c_Actinobacteria; o_Actinomycetales; f_Streptomycetaceae | 2.84 | **0.0177** |
| p_Proteobacteria; c_Gammaproteobacteria; o_Aeromonadales; f_Aeromonadaceae; g_Aeromonas | 3.39 | **0.0170** |
| p_Proteobacteria; c_Betaproteobacteria; o_Neisseriales; f_Neisseriaceae; g_Neisseria | 2.97 | **0.0138** |
| p_Proteobacteria; c_Gammaproteobacteria; o_Xanthomonadales; f_Xanthomonadaceae | 3.36 | **0.0029** |
| p_Proteobacteria; c_Betaproteobacteria; o_Burkholderiales; f_Oxalobacteraceae | 2.75 | **0.0048** |
| p_Actinobacteria; c_Actinobacteria; o_Actinomycetales; f_Micrococcaceae; g_Nesterenkonia | 2.68 | **0.0170** |
| p_Bacteroidetes; c_Sphingobacteriia; o_Sphingobacteriales; f_Sphingobacteriaceae; g_Pedobacter | 3.25 | **0.0170** |
| p_Firmicutes; c_Bacilli; o_Lactobacillales; f_Streptococcaceae; g_Lactococcus | 3.03 | **0.0001** |
| p_Proteobacteria; c_Alphaproteobacteria; o_Rhizobiales; f_Phyllobacteriaceae | 3.56 | **0.0412** |
| p_Proteobacteria; c_Epsilonproteobacteria; o_Campylobacterales; f_Helicobacteraceae; g_Flexispira | 3.46 | **0.0355** |
| p_Proteobacteria; c_Alphaproteobacteria; o_Rhizobiales; f_Beijerinckiaceae | 2.86 | **0.0077** |
| p_Thermotogae; c_Thermotogae; o_Thermotogales; f_Thermotogaceae; g_S1 | 3.49 | **0.0317** |
| p_Proteobacteria; c_Alphaproteobacteria; o_Rhodospirillales; f_Rhodospirillaceae | 2.95 | **0.0170** |
| p_Actinobacteria; c_Coriobacteriia; o_Coriobacteriales; f_Coriobacteriaceae; g_Collinsella | 3.31 | **0.0023** |
| 3xTg Male 5 months old | | |
| p_Firmicutes;c_Clostridia;o_Clostridiales;f_Christensenellaceae | 2.80 | **0.0042** |
| LDA: Linear discriminant analysis. The threshold on the logarithmic LDA score for discriminative features was set to 2.0 as indicated. “p” phylum, “c”, class; “o”, order; “f”, family; “g”, genus. The *p*-values were calculated using Kruskal-Wallis test. *p* ≤ 0.05 are considered statistically significant and are marked in bold font (see Figure 12). | | |
